# Genetic and observational evidence supports a causal role of sex hormones on the development of asthma

**DOI:** 10.1136/thoraxjnl-2018-212207

**Published:** 2019-04-01

**Authors:** Ryan Arathimos, Raquel Granell, Philip Haycock, Rebecca C Richmond, James Yarmolinsky, Caroline L Relton, Kate Tilling

**Affiliations:** 1 Population Health Sciences, Bristol Medical School, University of Bristol, Bristol, UK; 2 Medical Research Council Integrative Epidemiology Unit, University of Bristol, Bristol, UK

**Keywords:** sex hormones, cohort, asthma, testosterone, SHBG, ALSPAC

## Abstract

**Introduction:**

Males have a higher prevalence of asthma in childhood, whereas females have a higher prevalence in adolescence and adulthood. The ‘adolescent switch’ observed between sexes during puberty has been hypothesised to be due to fluctuating sex hormones. Robust evidence of the involvement of sex hormones in asthma could lead to development of therapeutic interventions.

**Methods:**

We combine observational evidence using longitudinal data on sex hormone-binding globulin (SHBG), total and bioavailable testosterone and asthma from a subset of males (n=512) in the Avon Longitudinal Study of Parents and Children, and genetic evidence of SHBG and asthma using two-sample Mendelian randomisation (MR), a method of causal inference. We meta-analysed two-sample MR results across two large data sets, the Trans-National Asthma Genetics Consortium genome-wide association study of asthma and UK Biobank (over 460 000 individuals combined).

**Results:**

Observational evidence indicated weak evidence of a protective effect of increased circulating testosterone on asthma in males in adolescence, but no strong pattern of association with SHBG. Genetic evidence using two-sample MR indicated a protective effect of increased SHBG, with an OR for asthma of 0.86 (95% CI 0.74 to 1.00) for the inverse-variance weighted approach and an OR of 0.83 (95% CI 0.72 to 0.96) for the weighted median estimator, per unit increase in natural log SHBG. A sex-stratified sensitivity analysis suggested the protective effect of SHBG was mostly evident in females.

**Conclusion:**

We report the first suggestive evidence of a protective effect of genetically elevated SHBG on asthma, which may provide a biological explanation behind the observed asthma sex discordance. Further work is required to disentangle the downstream effects of SHBG on asthma and the molecular pathways involved.

**Key messages**

**What is the key question?**

- Is there evidence for the involvement of sex hormones in the development of asthma that may explain the sex discordance in asthma prevalence?

**What is the bottom line?**

- Increased circulating sex hormone-binding globulin appears to be causally associated with a decreased risk of asthma, with effects stronger in females.

**Why read on?**

- This is the first study to investigate the effects of sex hormones on asthma and uses a dual observational and genetic epidemiological approach drawing on multiple independent data sets.

## Introduction

Observational epidemiological studies suggest that males have a higher prevalence of asthma in childhood, whereas females have a higher prevalence in adolescence and adulthood.^1 2^ The age at which this switch occurs has not been precisely determined, with conflicting research on whether puberty is associated with the switch in prevalence.^1 3^ The cause of the switch has been suggested to be multifactorial^4^ with sex hormones hypothesised to play a role in this switch, due to puberty coinciding with fluctuations in levels of circulating sex hormones.^5^

The hypothesised involvement of sex hormones in asthma is further supported by the acute deterioration of asthma observed during the menstrual cycle^6 7^ (coinciding with heightened sex hormone levels), as well as by the known gender dimorphism of the immune system^8^ (with females generally having stronger innate and adaptive immune responses than males in adulthood), and by the vast difference in prevalence of autoimmune disorders where over 75% of cases are female.^9^ In animal models, testosterone has been indicated to be immunosuppressant^10^ whereas oestrogen is potentially proinflammatory.^11^

Sex hormone-binding globulin (SHBG) is a glycoprotein and the major transporter and putative regulator of androgens (eg, testosterone) and oestrogens. SHBG may also exert direct effects of its own independently of the sex hormones it regulates.^12^ Variation in SHBG levels has been previously associated with risk of cancer^13^ and type 2 diabetes.^14^ The potential remains that the effects of sex hormones may be put to therapeutic use in the context of asthma. However, robust evidence of their involvement from human population studies is first required.

Observational epidemiological studies are often subject to confounding and reverse causation. Mendelian randomisation (MR) is a method of causal inference that uses germline genetic variants (usually single nucleotide polymorphisms (SNP)) as instrumental variables (IV).^15 16^ Based on Mendel’s law of independent assortment, genetic variants are distributed independently and randomly with respect to other genetic variants that are not in linkage disequilibrium and assuming no population stratification. Randomisation of genetic variants ensures that any association observed between the IVs and the disease outcome is most likely explained by an unbiased causal effect of the exposure on the outcome, given a set of prior assumptions.^16^ This is because MR study design is analogous to a randomised controlled trial with genetic variants randomising individuals to a higher or lower lifetime exposure to the trait of interest. In the case of SHBG as the exposure of interest, carriers of variant alleles associated with elevated circulating SHBG in previous genome-wide association studies (GWAS) may be considered as being genetically randomised to higher lifetime average levels of circulating SHBG. MR application is increasingly facilitated by the availability of summary statistics from GWAS from large consortia, with IV-exposure estimates and IV-outcome estimates derived from different data sets, in an approach termed two-sample MR.^17^

Both SHBG and testosterone levels are highly heritable traits with previous GWAS identifying multiple SNPs associated with both traits.^18 19^ The strongest association has been observed between the rs12150660 SNP located in the *SHBG* gene and circulating SHBG (p=1.8×10^−106^), estimated to account for ~7.8% of circulating SHBG variation in males and ~3.3% in females, in a study with a combined discovery plus replication sample size of 28 837 individuals.^18^ SNPs identified as associated with circulating testosterone largely overlap those identified for SHBG.

In this study, we test the hypothesis that circulating sex hormones have an effect on development of asthma. We combine observational evidence using longitudinal sex hormone data from a subset of males in the Avon Longitudinal Study of Parents and Children (ALSPAC) cohort, with genetic evidence from two-sample MR in two large data sets, the Trans-National Asthma Genetics Consortium (TAGC) GWAS of asthma and UK Biobank.

## Methods

### Study populations

#### Avon Longitudinal Study of Parents and Children

The ALSPAC recruited 14 541 pregnant women resident in Avon, UK with expected dates of delivery from 1 April 1991 to 31 December 1992.^20 21^ A detailed description of the cohort can be found in online supplementary methods appendix A1. SHBG and total testosterone (TT) were measured in a subset of 513 males at 9.9, 11.8, 13.8, 15.5 and 17.8 years of age using ELISA in peripheral blood samples as previously described.^22^ Longitudinal sex hormone measurements were not available for females in ALSPAC. Bioavailable testosterone (BT) was derived from measures of TT and SHBG as previously described.^22^ Genetic data for the ALSPAC children were generated by Sample Logistics and Genotyping Facilities at the Wellcome Trust Sanger Institute and LabCorp. Data on asthma in the last 12 months were extracted from questionnaires completed by the ALSPAC mothers at 10.7, 13.1 and 13.8 years of age, and by the study participants themselves at 16.5 and 22.9 years. Details of the hormone measurements, genotype preprocessing and derived asthma measures can be found in online supplementary methods appendix A1.

10.1136/thoraxjnl-2018-212207.supp1 Supplementary data

#### UK Biobank

The UK Biobank project is a large prospective cohort study of approximately 500 000 individuals from across the UK, aged 40–69 years at recruitment. Details of the study recruitment have been previously described.^23 24^ Genotype imputation and quality control (QC) are described in online supplementary methods appendix A1. Data on ever having had an asthma diagnosis were self-reported by UK Biobank participants by touchscreen questionnaire. Following genotype-related exclusions and QC, a total of 38 337 asthma cases and 296 245 controls remained.

#### Trans-National Asthma Genetics Consortium genome-wide association study

The TAGC performed a large-scale ancestry-specific GWAS of asthma^25^ using a total of 23 948 asthma cases and 118 538 controls from 66 studies. The subsample of European ancestry comprised 19 954 asthma cases and 107 715 controls from 56 studies, identifying 673 genome-wide significant SNPs. The study meta-analysed results from cohorts of individuals with both childhood-onset and adult-onset asthma across a range of ages. Full summary statistics from the GWAS meta-analysis for all analysed SNPs are available online via the GWAS catalogue.^26^ The results of the random effects meta-analysis of European ancestry were used in all analyses.

### Statistical analyses

#### Observational analyses

Cross-sectional associations of sex hormones and asthma at each of the five time points in ALSPAC were investigated using serial logistic regression in STATA V.14.2. Model 1 was unadjusted for the previous measure of asthma/hormone and model 2 was adjusted for the previous measure, therefore modelling change between time points. Hormone measures were matched with their closest asthma response, ensuring asthma (the outcome) proceeded the hormone measurement. Analyses were carried out in male singletons (n=512). Results of cross-sectional models were compared with the path analysis.

In order to test for potential differences in effects between time points, path analysis (a type of multiple regression analysis) was conducted using STATA V.14.2 and STATA structural equation model (SEM) builder. Separate generalised SEMs were fitted for SHBG, TT and BT and asthma (online supplementary figures S1–S3 appendix A1). All models were adjusted for maternal confounders (maternal smoking, maternal education, parity, gestational age, maternal age) as well as participants’ exact age. Details of covariate adjustments and the rationale of the path analyses can be found in online supplementary methods appendix A1.

In order to increase statistical power and counter possible bias due to missingness, multiple imputation was used to complete missing values in the exposure, outcome and covariates, in both the cross-sectional regressions and the path analysis using ICE^27^ in STATA V.14.2 (StataCorp, release 14).^27^ A detailed description of the multiple imputation can be seen in online supplementary appendix A1. Results using the imputed data set were compared with the complete case analysis. Descriptions of the variables are shown in table 1.

**Table 1:** Descriptions of the different sex hormone, asthma and confounder variables included in the observational analyses, with percentage missingness in the original variables out of a total subsample size of 512 males in the ALSPAC cohort

Age at measurement	n	Mean (SD)	Missing (%)*	Imputation method
SHBG				
9.9	441	92.33 (43.67)	13.9	PMM
11.8	482	74.96 (37.47)	5.9	PMM
13.8	416	45.41 (26.03)	18.8	PMM
15.5	463	30.82 (13.33)	9.6	PMM
17.8	427	26.26 (12.39)	16.6	PMM
Total testosterone				
9.9	440	0.82 (0.09)	14.1	PMM
11.8	481	1.54 (1.23)	6.1	PMM
13.8	414	9.05 (4.66)	19.1	PMM
15.5	463	14.8 (2.72)	9.6	PMM
17.8	412	16.48 (2.62)	19.5	PMM
Bioavailable testosterone				
9.9	441	0.17 (0.07)	14.1	PMM
11.8	482	0.42 (0.47)	6.1	PMM
13.8	414	3.84 (2.57)	19.1	PMM
15.5	464	7.33 (1.97)	9.6	PMM
17.8	413	9.01 (1.97)	19.5	PMM
Asthma				
10.7	467	–	8.8	Logit
13.1	476	–	7.0	Logit
13.8	455	–	11.1	Logit
16.5	473	–	7.6	Logit
22.9	250	–	51.2	Logit
Maternal confounders				
Maternal education	477	–	6.8	Mlogit
Maternal smoking	481	–	6.1	Mlogit
Parity	474	–	7.4	Logit
Maternal age	492	363.52 (52.52)	3.9	Regress
Gestational age	488	39.42 (1.86)	4.7	Regress

*As a percentage of total size of male subsample that had at least two hormone measures at two time points (n=512). Missingness estimate does not take into account non-overlap between asthma responses and hormone measurements. For example, although there are n=440 with testosterone at 9.9 years and n=467 with asthma data at 10.7, individuals with data do not fully overlap.

ALSPAC, Avon Longitudinal Study of Parents and Children; Logit, logistic regression; Mlogit, multinomial logistic regression; PMM, predictive mean matching; Regress, linear regression; SHBG, sex hormone-binding globulin.

### Genetic analyses

#### Age-dependent effects of SHBG variants

As a preliminary step, we examined whether the effects of the SNPs associated with SHBG in the GWAS by Coviello *et al* 18 had an age-dependent effect on SHBG and TT using the ALSPAC subsample (males aged 9.9–17.8 years). Generalised linear regression models were implemented in R V.3.4.1 to test cross-sectional associations between each of the SNPs and either z-scored SHBG, TT or BT at each of the five time points, adjusting for the top 20 derived principal components (PC).

#### Mendelian randomisation

Two-sample MR was used to investigate the hypothesised causal effect of SHBG on asthma. The two-sample approach requires only summary-level data from GWAS, enabling SNP-outcome and SNP-exposure effects to be derived from different data sets. The assumptions of two-sample MR are similar to those of one-sample MR, while additionally requiring that the summary statistics for both the exposure and the outcome are derived from studies of comparable populations. Analyses were performed using the TwoSampleMR R package, part of MR-Base.^17^ The inverse-variance weighted (IVW) approach was used as a primary analysis, with two complementary estimation methods as sensitivity analyses to assess horizontal pleiotropy (where the genetic variant associates with the outcome via an independent pathway to the exposure): a weighted-median estimator^28^ and MR Egger regression.^29^ Evidence for effect size dilution was assessed using the unweighted I^2^ statistic,^30^ also called the I^2^_GX_ statistic.

##### SNP-asthma effects

Individual-level data from UK Biobank and summary statistics from the TAGC GWAS of asthma (where ALSPAC was a contributing cohort) were used to derive SNP-asthma effects. In UK Biobank logistic regressions in R 3.4.1 were used to determine the effects of each SNP on self-reported ever asthma, adjusted for genotype array (chip), sex and top 40 PCs. For TAGC, the results of the random effects meta-analysis of European ancestry were used.

##### IV combinations

**Figure 1:**
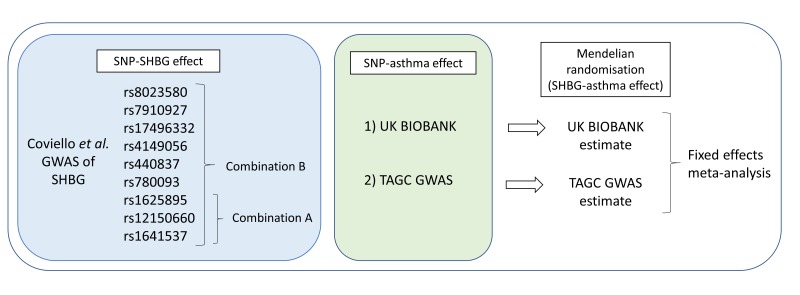
Flow diagram of the Mendelian randomisation (MR) analyses performed. GWAS, genome-wide association studies; SHBG, sex hormone-binding globulin; SNP, single nucleotide polymorphism; TAGC, Trans-National Asthma Genetic Consortium.

Two combinations of independent SHBG IVs were used, based on associations reported in the previous GWAS (online supplementary table S5)^18^. IV combination A comprised entirely SNPs located in the *SHBG* gene, determined as independent variants in the conditional analysis by Coviello *et al*.^18^ Variants in the *SHBG* gene region are less likely to be subject to horizontal pleiotropy. This resulted in three variants comprising IV combination A. For IV combination B, all SNPs identified in the previous GWAS of SHBG were used (nine variants), including those in combination A. We excluded only SNPs located on the X-chromosome (rs1573036), SNPs with a minor allele frequency <0.01 in UK Biobank (rs6258), or SNPs unavailable in the Haplotype Reference Consortium-imputed UK Biobank data set (rs2411984). This approach using two complementary IV combinations has been used previously for assessing the effects of SHBG on metabolites^31^ in an MR framework. The diagram in figure 1 presents an overview of the analyses performed.

##### Meta-analysis

Final MR estimated effects of SHBG on asthma resulting from both the TAGC GWAS and UK Biobank were combined in a fixed effects meta-analysis using the metafor R package. The combined meta-analysis sample size consisted of 462 251 individuals of which 58 291 were asthma cases.

##### Sex-stratified analysis

Since we hypothesised that the effects of SHBG were likely to be sex specific, MR analyses were repeated on a sex-stratified sample in UK Biobank, separately for male (n=154 564 of which 16 308 asthma cases) and female participants (n=180 018 of which 22 029 asthma cases).

## Results

### Observational analyses

Descriptions of the different sex hormone measurements, asthma reports and confounder variables included in the observational analyses, with percentage missingness for each variable, are presented in table 1.

Serial cross-sectional models of SHBG, TT and BT in males revealed little evidence of an association between SHBG and asthma (model 1 in online supplementary table S1 appendix A1), but a consistent inverse association between TT and BT on asthma after 11.8 years (model 1 in online supplementary tables S2 and S3 appendix A1). However, CIs were wide for both TT and BT and overlapped the null. Serial cross-sectional models using multiple imputation and adjusted for previous measures of asthma and either SHBG, TT or BT (table 2) demonstrated similar estimates to the base models. For SHBG there was an OR for asthma of 0.96 (95% CI 0.74 to 1.24), 1.54 (95% CI 0.71 to 3.37), 1.32 (95% CI 0.64 to 2.70), 1.18 (95% CI 0.67 to 2.06) and 1.02 (95% CI 0.57 to 1.83) at 9.9, 11.8, 13.8, 15.5 and 17.8 years, respectively. For TT there was an OR for asthma of 1.12 (95% CI 0.87 to 1.43), 0.85 (95% CI 0.55 to 1.32), 0.80 (95% CI 0.46 to 1.38), 0.89 (95% CI 0.59 to 1.35) and 0.71 (95% CI 0.43 to 1.16) at the respective time points, whereas for BT there was an OR of 1.02 (95% CI 0.77 to 1.34), 0.79 (95% CI 0.47 to 1.34), 0.86 (95% CI 0.48 to 1.55), 0.87 (95% CI 0.56 to 1.35) and 0.87 (95% CI 0.54 to 1.41) at the respective time points. Comparison of the different cross-sectional models can be seen in online supplementary tables S1–S3 appendix A1.

**Table 2:** Results of serial cross-sectional analysis of SHBG, TT or BT on asthma (using multiple imputation) adjusted for previous measures of asthma and sex hormones (where available) at five time points in a subsample of 512 males in ALSPAC

Age at sex hormone exposure (years)	Age at asthma outcome (years)	OR* (95% CI) SHBG	n with data for SHBG (% missing)†	OR* (95% CI) total testosterone	n with data for total testosterone (% missing)†	OR* (95% CI) bioavailable testosterone	n with data for bioavailable testosterone (% missing)	n after multiple imputation
9.9	10.7	0.96 (0.74 to 1.24)	381 (26)	1.12 (0.87 to 1.43)	381 (26)	1.02 (0.77 to 1.34)	381 (26)	512
11.8	13.1	1.54 (0.71 to 3.37)	339 (34)	0.85 (0.55 to 1.32)	338 (34)	0.79 (0.47 to 1.34)	338 (34)	512
13.8	13.8	1.32 (0.64 to 2.70)	318 (38)	0.80 (0.46 to 1.38)	315 (38)	0.86 (0.48 to 1.55)	315 (38)	512
15.5	16.5	1.18 (0.67 to 2.06)	263 (49)	0.89 (0.59 to 1.35)	262 (49)	0.87 (0.56 to 1.35)	262 (49)	512
17.8	22.9	1.02 (0.57 to 1.83)	144 (72)	0.71 (0.43 to 1.16)	99 (81)	0.87 (0.54 to 1.41)	99 (81)	512

*OR for asthma per SD increase in either SHBG, TT or BT.

†n refers to the number of individuals with complete data in the analysis of specified hormone (before multiple imputation). Per cent missing refers to the fraction of individuals missing some data (either asthma measurements, hormone measurements or covariates, where covariates include any previous measurement of asthma and hormone) at each time point, which were subject to multiple imputation.

ALSPAC, Avon Longitudinal Study of Parents and Children; BT, bioavailable testosterone; SHBG, sex hormone-binding globulin; TT, total testosterone.

Results of path analyses using multiple imputation (online supplementary table S4 and figures S1–S3 appendix A1) were similar to results from the serial cross-sectional models, with no clear evidence of an association between SHBG and asthma and only weak evidence for a protective effect of increased TT on asthma in adolescence (table 2). Since there was little evidence of an association at the individual time points in either the serial cross-sectional or the path analysis we did no test for differences between time points in the path analysis. We therefore present the results of the simpler cross-sectional models in table 2, with the results of the path analysis in online supplementary table S4 appendix A1.

### Genetic analyses

#### Age-dependent effects of SHBG variants

**Figure 2:**
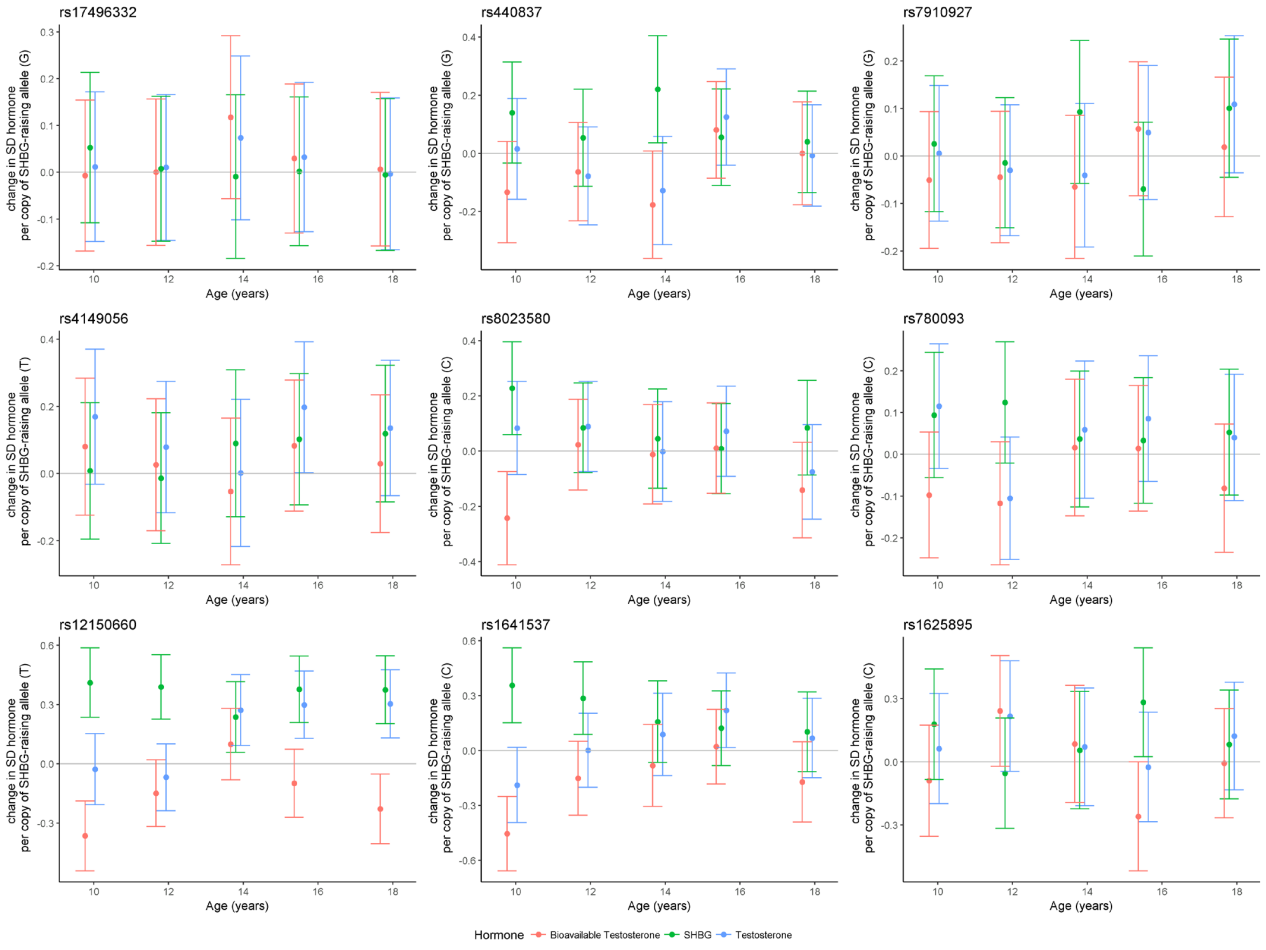
Serial cross-sectional associations of estimated SD change in SHBG, bioavailable testosterone (BT) or total testosterone (TT), per copy of SHBG-raising allele for nine genetic variants used as SHBG instrumental variables (IV) in the Mendelian randomisation (MR) analyses. Associations assessed in a subset of Avon Longitudinal Study of Parents and Children (ALSPAC) males across five available time points. SHBG, sex hormone-binding globulin.

Serial cross-sectional analysis of the effect of the SHBG SNPs in ALSPAC revealed that only the rs12150660 variant had a consistent effect on SHBG levels and an age-dependent effect on TT and BT (figure 2 and online supplementary appendix A2). For SHBG there was an estimated 0.41 (95% CI 0.24 to 0.59), 0.39 (95% CI 0.23 to 0.55), 0.24 (95% CI 0.06 to 0.42), 0.38 (95% CI 0.21 to 0.55) and 0.38 (95% CI 0.20 to 0.55) SD increase in circulating SHBG (nmol/L) per copy of the rs12150660 variant allele (GT or TT genotype) at the ages of 9.9, 11.8, 13.8, 15.5 and 17.8 years, respectively. For TT, there was no evidence of an association at the earlier ages, however an effect was observed at the later time points. There was an estimated −0.03 (95% CI −0.21 to 0.15), −0.07 (95% CI −0.24 to 0.10), 0.27 (95% CI 0.09 to 0.45), 0.30 (95% CI 0.13 to 0.47) and 0.30 (95% CI 0.13 to 0.48) SD increase in testosterone per copy of the rs12150660 variant allele at the ages of 9.9, 11.8, 13.8, 15.5 and 17.8 years, respectively. For BT, there was an inverse pattern of association to that of SHBG, consistent with its derivation from the assayed SHBG and TT measures. There was a −0.37 (95% CI −0.54 to −0.19), −0.15 (95% CI −0.32 to 0.02), 0.10 (95% CI −0.09 to 0.28), −0.10 (95% CI −0.27 to 0.07) and −0.23 (95% CI −0.40 to −0.05) SD change in BT per copy of the rs12150660 variant allele at the five respective time points. There was also some evidence for an effect of rs1641537 and rs8023580 on SHBG and BT levels in childhood (figure 2 and online supplementary appendix A2). However, the remaining SNPs indicated inconsistent patterns of effect on SHBG, TT and BT across the five time points.

10.1136/thoraxjnl-2018-212207.supp2 Supplementary data

#### Mendelian randomisation

**Figure 3:**
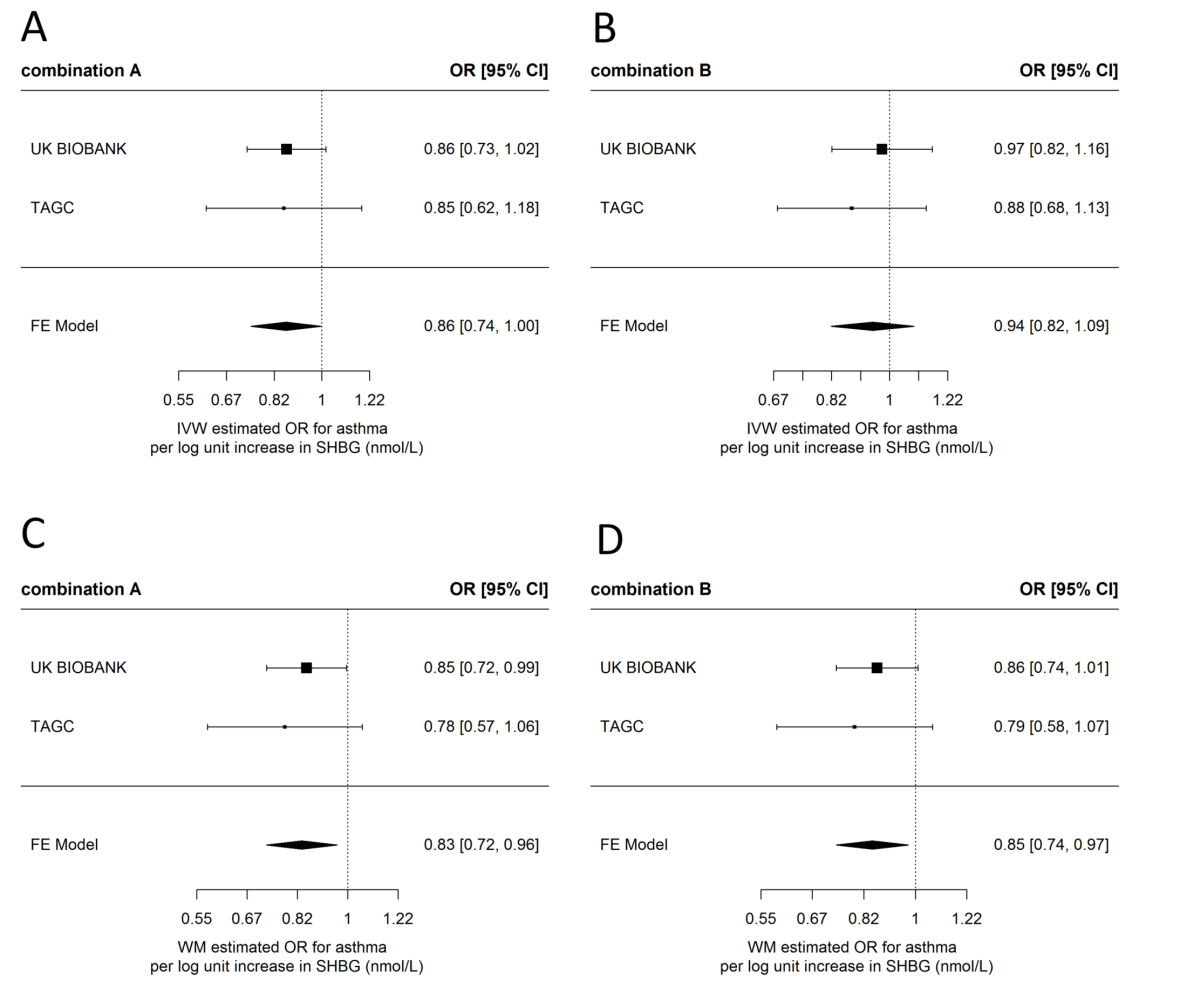
Meta-analysis forest plots of the estimated effects of the two SHBG instrumental variable (IV) combinations on asthma, in the TAGC genome-wide association studies (GWAS) and UK Biobank. Fixed effects  (FE)  meta-analysis of (A) the IVW estimated effect of IV combination A on asthma, (B) the IVW estimated effect of IV combination B on asthma, (C) the weighted-median (WM) estimated effect of IV combination A on asthma, (D) the WM estimated effect of IV combination B on asthma. IVW, inverse-variance weighted; SHBG, sex hormone-binding globulin; TAGC, Trans-National Asthma Genetic Consortium.

In contrast to the observational findings, there was evidence of a protective effect of increased SHBG on asthma when using MR (figure 3 and online supplementary tables S6-S7 and supplementary table S8 appendix A1). In IVW analyses, there was an estimated 0.86 (95% CI 0.74 to 1.00) OR per unit increase in log SHBG for IV combination A and 0.94 (95% CI 0.82 to 1.09) OR for asthma when using IV combination B. Meta-analysed weighted-median estimates were similar, with a 0.83 (95% CI 0.72 to 0.96) OR for asthma per unit increase in log SHBG for IV combination A and an estimated 0.85 (95% CI 0.74 to 0.97) OR for asthma for IV combination B. Wider CIs and smaller effect sizes in the IVW estimated effects of combination B may indicate the presence of directional pleiotropy, which the IVW method is not robust to. This conclusion is supported by the results of the more conservative combination A, which is less likely to introduce pleiotropic effects. Estimates from MR Egger regression for combination B (MR Egger regression was not applied to combination A as there were not enough SNPs) were in the same direction as estimates from the other two methods with a 0.81 (95% CI 0.60 to 1.09) OR for asthma per unit increase in log SHBG, but with wider CIs that spanned the null (online supplementary figure S4 appendix A1), possibly reflecting the lower statistical power of MR Egger regression. There was no indication of dilution in the MR Egger regression estimates (I^2^ for dilution=0.99). Heterogeneity tests for combination B in UK Biobank indicated some evidence for heterogeneity between SNPs for the IVW method (Cochran’s Q=15.4, Q p=0.051), with an I^2^ for heterogeneity between SNPs of 48.1%. There was weak evidence for directional pleiotropy in combination B in UK Biobank, estimated using the MR Egger intercept (Egger intercept=1.01, p=0.173). Scatterplots of the SNP-asthma and SNP-SHBG effects can be seen in online supplementary figures S5 and S6 appendix A1.

##### Sex-stratified MR analysis

**Figure 4:**
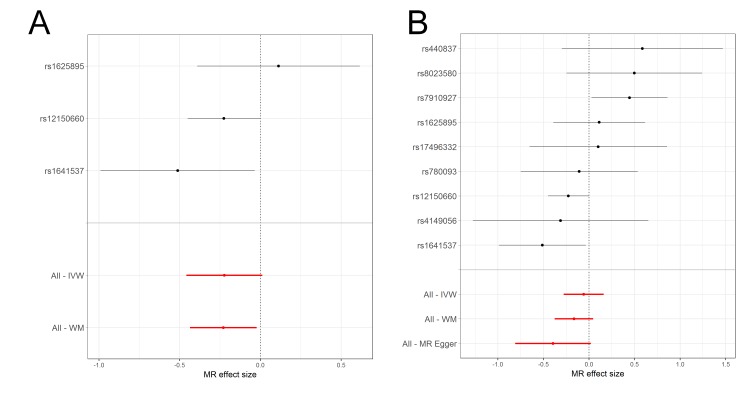
Forest plots of the individual instrumental variable (IV) effects in the female subsample of UK Biobank using the two combinations of IVs to assess the genetic effect of sex hormone-binding globulin (SHBG) on asthma. MR effect sizes are presented as log odds for asthma per unit increase in log SHBG. Methods used include the weighted-median (All-WM), MR Egger regression (All-MR Egger) and inverse-variance weighted (All-IVW). (A) IV combination A includes nine single nucleotide polymorphisms (SNP) and (B) IV combination B includes three SNPs located in the SHBG gene. MR, Mendelian randomisation.

**Figure 5:**
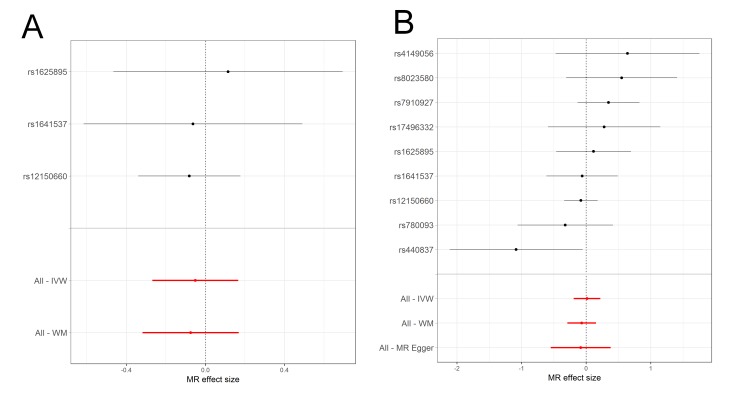
Forest plots of the individual instrumental variable (IV) effects in the male subsample of UK Biobank using the two combinations of IVs to assess the genetic effect of sex hormone-binding globulin (SHBG) on asthma. Mendelian randomisation (MR) effect sizes are presented as log odds for asthma per unit increase in log SHBG. Methods used include the weighted-median (All-WM), MR Egger regression (All-MR Egger) and inverse-variance weighted (All-IVW). (A) IV combination A includes nine single nucleotide polymorphisms (SNP) and (B) IV combination B includes three SNPs located in the SHBG gene.

The sex-stratified analysis in UK Biobank indicated differences in MR estimated effects between females and males (figure 4 and figure 5). For females, there was an IVW estimated 0.80 (95% CI 0.63 to 1.01) OR for asthma per unit increase in log SHBG when using IV combination A, whereas in males there was an IVW estimated 0.95 (95% CI 0.77 to 1.18) OR for asthma. For the weighted-median estimated effect there was an OR of 0.80 (95% CI 0.64 to 0.98) for females and an OR of 0.93 (95% CI 0.73 to 1.17) for males. When using IV combination B, similar evidence for a protective effect of SHBG on asthma was obtained in females, whereas these estimates largely agreed with estimates based on IV combination A in males. Larger effects were observed in females when using the weighted-median estimator and MR Egger regression sensitivity analyses than the IVW estimate, indicating horizontal pleiotropy in IV combination B (complete description of results in online supplementary tables S9-S10 and supplementary result appendix A1).

## Discussion

We have conducted a novel hybrid investigation of the associations of SHBG and testosterone with asthma using dual longitudinal data analysis and an MR design. Observational evidence indicated some evidence of a protective effect of increased TT on asthma, but no clear pattern for SHBG. Genetic evidence using two-sample MR indicated a protective effect of increased SHBG on asthma, with the effects most evident among females and indications of horizontal pleiotropy in the more liberal combination of IVs.

Notably, the sole genetic variant to demonstrate a consistent effect on circulating SHBG in ALSPAC was the rs12150660 SNP. However, there was no discernible effect of the variant on TT levels until puberty, gradually increasing during teenage years. This observation fits with previously reported biological understanding of the polymorphism, suggesting that its location near a pentanucleotide repeat which affects SHBG expression levels in vitro^32^ drives the association. The remaining eight SNPs showed inconsistent patterns of association with circulating SHBG. However, associations may not have replicated because of lack of statistical power due to the small sample size or because effects of these SNPs may not be evident until later life (the original GWAS included only adult participants).

Although previous hypotheses have suggested an immunological/inflammatory effect of SHBG on asthma, MR does not distinguish between which mechanistic pathways link the exposure to the outcome. Therefore, any indication of SHBG’s causality does not imply a biological or immunological effect of sex hormones.

The differences in effects of SHBG on asthma between sexes observed have the potential to explain the increase in female asthmatics observed after puberty. Since the evidence indicates that an increase in SHBG is protective against asthma, then the decrease in circulating SHBG observed in females during puberty^33 34^ may be a biological trigger for development of asthma.

Little previous evidence exists of the effects of sex hormones on asthma. We have not been able to find any previous literature that has investigated the effect of circulating SHBG on asthma that could be used to facilitate comparisons with our MR estimated effects.

### Strengths and limitations

The current study has several major strengths. It is based on strong biological rationale with a hypothesis that has been extensively reviewed.^35^ The biological hypothesis is underpinned by an established observation of sex discordance in asthma prevalence, previously corroborated in ALSPAC.^36^ The current analyses combine observational evidence with genetic evidence from MR, using a triangulation approach,^37^ and multiple complementary approaches and data sets, strengthening the conclusions.

#### Observational analyses

In the observational analysis of the ALSPAC subsample, testosterone distribution at 13.8 years was severely non-normal which may lead to imprecision of the SEs of the estimated effects. It was hypothesised that this was due to some but not all males having transitioned through puberty. However, since the non-normality is observed only at the one time point, estimates from this single measure do not alter the conclusions.

All male hormone measurements were carried out using immunoassays, with evidence suggesting that immunoassays lack precision, particularly in children, where testosterone levels are low.^38^ At the earliest time point, a large number of measurements had to be set to the lowest detectable assay value. Sample sizes were also small due to the non-overlap of individuals with reports of asthma and those with measured sex hormones, with a large fraction of missing measurements, particularly at the final time point (table 1). We used multiple imputation to attempt to increase power for the analysis and additionally included reports of wheezing in the last 12 months as auxiliary variables that may predict asthma in order to aid the multiple imputation. Assuming an adequate number of imputations and valid imputation, that is, all data are missing at random and the imputation and analysis models are correct, the results from multiple imputation should be unbiased. However, multiple imputation will lead to large SEs and wide CIs if there is little overall information in the data sets. After multiple imputation, we observed large SEs and wide CIs in our estimated effects, indicating that statistical power to detect an association may have remained low in the sample size of 512 individuals despite imputation. All observational analyses were conducted in a subsample of males, and the observational effects of SHBG and testosterone on asthma in females have not been assessed, with the effects likely to differ substantially from males given the differences in effects between the two sexes observed in the genetic analyses.

#### Genetic analyses

A major limitation of the genetic analyses is that a two-sample MR-based approach estimates average lifetime effects of an exposure on a disease outcome and does not distinguish between changes in the magnitude of that effect during different critical periods of the life course. This means that overall effect estimates may not be representative of true effects at all ages if the exposure only affects the outcome at certain critical periods of the life course. This is particularly true for diseases such as asthma which are known to wax and wane. It was therefore not possible to determine if SHBG is causal to the ‘adolescent switch’ observed in asthma prevalence or if the decreased risk is an unrelated early-life or later-life effect of SHBG on asthma. Future studies could attempt to develop methods to investigate time-varying exposures (eg, SHBG) on time-varying outcomes (eg, asthma) in an MR framework.

We did not attempt to stratify the MR analysis by age of asthma onset due to measurement error being a potential major problem in the UK Biobank, as the adult participants aged 40–69 at recruitment were asked to recall age of asthma onset, likely leading to substantial imprecision in reports. Additionally, as SNPs used as SHBG IVs in the MR were derived from adult population GWAS, we considered them unlikely to proxy SHBG levels as robustly in childhood, given the results of the SNP validation step in the ALSPAC subsample (shown in figure 2), which would further reduce statistical power to detect an association. Similarly, although the TAGC GWAS consortium performed a sensitivity analysis where participants were stratified into childhood-onset asthma and adult-onset asthma, the cut-off age for childhood-onset asthma was 16 years of age, by which time any changes linked to sex hormones will have already occurred. Therefore, we did not conduct an age-of-onset stratified MR to compare the effects of sex hormones prepuberty versus postpuberty. In the sex-stratified analysis in UK Biobank, the lower number of asthma cases in the male subsample compared with the female subsample may have led to lower statistical power and wider CIs, which may explain the differences in results. Given that the CIs overlap substantially, we cannot determine with certainty whether effects are specific to females.

The genetic correlation between SHBG and testosterone (as well as other hormones such as oestrogens) presents a problem in MR and it was not possible to identify SNPs associated with testosterone that were not also associated with SHBG. A previous GWAS of testosterone^19^ identified several polymorphisms associated with testosterone, including the rs12150660 variant; however, all reported variants were also identified as associated with SHBG. It may be possible to investigate such situations in the future using multivariate MR.^39^ Winner’s curse is a likely issue in two-sample MR studies that derive SNP-exposure estimates from the same GWAS used to select IVs, and although we used effect estimates from the combined discovery and replication samples of the published GWAS of SHBG, which may mitigate effects due to winner’s curse, some residual bias may remain.

Overall, we have performed multiple analyses at several different time points in order to disentangle the complex relationship between the various sex hormones and asthma. The possibility of spurious results arising due to chance cannot therefore be ruled out. Future work should attempt to replicate these findings in independent data sets in order to confirm the current conclusions.

## Conclusion

In this study, we present the first tentative evidence that increased circulating SHBG is causally associated with a decreased risk of asthma, with effects stronger in females. Although findings from the observational epidemiological analysis indicated little evidence of an effect of SHBG on asthma and only weak evidence for a protective effect of increased testosterone on asthma in the subsample of males, genetic epidemiological approach indicated some evidence of a protective effect of increased SHBG on asthma. These findings from multiple independent data sets align with previous hypotheses suggesting that fluctuating sex hormones, particularly during puberty, may promote asthma development in females. These suggestive associations may provide some insight into the biological mechanisms behind the observed asthma sex discordance as well as the overall pathology of asthma. Whether the effects of sex hormones are meaningful as potential intervention targets is unknown and further work is required to disentangle the downstream effects of SHBG on asthma.

## References

[1] Vink NM, Postma DS, Schouten JP, et al Gender differences in asthma development and remission during transition through puberty: the TRacking Adolescents' Individual Lives Survey (TRAILS) study. J Allergy Clin Immunol 2010;126:498–504. 10.1016/j.jaci.2010.06.018 20816186

[2] Strachan DP, Butland BK, Anderson HR Incidence and prognosis of asthma and wheezing illness from early childhood to age 33 in a national British cohort. BMJ 1996;312:1195–9. 10.1136/bmj.312.7040.1195 8634562 PMC2350975

[3] Fu L, Freishtat RJ, Gordish-Dressman H, et al Natural progression of childhood asthma symptoms and strong influence of sex and puberty. Ann Am Thorac Soc 2014;11:939–44. 10.1513/AnnalsATS.201402-084OC 24896645 PMC4213994

[4] Postma DS Gender differences in asthma development and progression. Gend Med 2007;4:S133–46. 10.1016/S1550-8579(07)80054-4 18156099

[5] Osman M Therapeutic implications of sex differences in asthma and atopy. Arch Dis Child 2003;88:587–90. 10.1136/adc.88.7.587 12818904 PMC1763154

[6] Tan KS, McFarlane LC, Lipworth BJ Modulation of airway reactivity and peak flow variability in asthmatics receiving the oral contraceptive pill. Am J Respir Crit Care Med 1997;155:1273–7. 10.1164/ajrccm.155.4.9105066 9105066

[7] Eliasson O, Scherzer HH, DeGraff AC Morbidity in asthma in relation to the menstrual cycle. J Allergy Clin Immunol 1986;77(1 Pt 1):87–94. 10.1016/0091-6749(86)90328-3 3944377

[8] Klein SL, Flanagan KL Sex differences in immune responses. Nat Rev Immunol 2016;16:626–38. 10.1038/nri.2016.90 27546235

[9] Jacobson DL, Gange SJ, Rose NR, et al Epidemiology and estimated population burden of selected autoimmune diseases in the United States. Clin Immunol Immunopathol 1997;84:223–43. 10.1006/clin.1997.4412 9281381

[10] Roberts M, Peters A Is testosterone immunosuppressive in a condition-dependent manner? An experimental test in blue tits. J Exp Biol 2009;212(Pt 12):1811–8. 10.1242/jeb.031047 19482998

[11] Straub RH The complex role of estrogens in inflammation. Endocr Rev 2007;28:521–74. 10.1210/er.2007-0001 17640948

[12] Hryb DJ, Khan MS, Romas NA, et al The control of the interaction of sex hormone-binding globulin with its receptor by steroid hormones. J Biol Chem 1990;265:6048–54. 2156840

[13] Gann PH, Hennekens CH, Ma J, et al Prospective study of sex hormone levels and risk of prostate cancer. J Natl Cancer Inst 1996;88:1118–26. 10.1093/jnci/88.16.1118 8757191

[14] Birkeland KI, Hanssen KF, Torjesen PA, et al Level of sex hormone-binding globulin is positively correlated with insulin sensitivity in men with type 2 diabetes. J Clin Endocrinol Metab 1993;76:275–8. 10.1210/jcem.76.2.8432768 8432768

[15] Smith GD, Ebrahim S ’Mendelian randomization': can genetic epidemiology contribute to understanding environmental determinants of disease? Int J Epidemiol 2003;32:1–22. 10.1093/ije/dyg070 12689998

[16] Davey Smith G, Hemani G Mendelian randomization: genetic anchors for causal inference in epidemiological studies. Hum Mol Genet 2014;23:R89–R98. 10.1093/hmg/ddu328 25064373 PMC4170722

[17] Hemani G, Zheng J, Wade KH, et al MR-Base: a platform for systematic causal inference across the phenome using billions of genetic associations. bioRxiv 2016.

[18] Coviello AD, Haring R, Wellons M, et al A genome-wide association meta-analysis of circulating sex hormone-binding globulin reveals multiple Loci implicated in sex steroid hormone regulation. PLoS Genet 2012;8:e1002805 10.1371/journal.pgen.1002805 22829776 PMC3400553

[19] Ohlsson C, Wallaschofski H, Lunetta KL, et al Genetic determinants of serum testosterone concentrations in men. PLoS Genet 2011;7:e1002313 10.1371/journal.pgen.1002313 21998597 PMC3188559

[20] Fraser A, Macdonald-Wallis C, Tilling K, et al Cohort profile: the Avon Longitudinal Study of Parents and Children: ALSPAC mothers cohort. Int J Epidemiol 2013;42:97–110. 10.1093/ije/dys066 22507742 PMC3600619

[21] Boyd A, Golding J, Macleod J, et al Cohort Profile: the ’children of the 90s'--the index offspring of the Avon Longitudinal Study of Parents and Children. Int J Epidemiol 2013;42:111–27. 10.1093/ije/dys064 22507743 PMC3600618

[22] Khairullah A, Klein LC, Ingle SM, et al Testosterone trajectories and reference ranges in a large longitudinal sample of male adolescents. PLoS One 2014;9:e108838 10.1371/journal.pone.0108838 25268961 PMC4182562

[23] Bycroft C, Freeman C, Petkova D, et al Genome-wide genetic data on ~500,000 UK Biobank participants. bioRxiv 2017.

[24] Sudlow C, Gallacher J, Allen N, et al UK biobank: an open access resource for identifying the causes of a wide range of complex diseases of middle and old age. PLoS Med 2015;12:e1001779 10.1371/journal.pmed.1001779 25826379 PMC4380465

[25] Demenais F, Margaritte-Jeannin P, Barnes KC, et al Multiancestry association study identifies new asthma risk loci that colocalize with immune-cell enhancer marks. Nat Genet 2018;50:42–53. 10.1038/s41588-017-0014-7 29273806 PMC5901974

[26] Welter D, MacArthur J, Morales J, et al The NHGRI GWAS Catalog, a curated resource of SNP-trait associations. Nucleic Acids Res 2014;42:D1001–6. 10.1093/nar/gkt1229 24316577 PMC3965119

[27] Royston P ICE: Stata module for multiple imputation of missing values. Statistical Software Components 2014.

[28] Bowden J, Davey Smith G, Haycock PC, et al Consistent estimation in mendelian randomization with some invalid instruments using a weighted median estimator. Genet Epidemiol 2016;40:304–14. 10.1002/gepi.21965 27061298 PMC4849733

[29] Bowden J, Davey Smith G, Burgess S Mendelian randomization with invalid instruments: effect estimation and bias detection through Egger regression. Int J Epidemiol 2015;44:512–25. 10.1093/ije/dyv080 26050253 PMC4469799

[30] Bowden J, Del Greco M F, Minelli C, et al Assessing the suitability of summary data for two-sample Mendelian randomization analyses using MR-Egger regression: the role of the I2 statistic. Int J Epidemiol 2016;45:1961–74. 10.1093/ije/dyw220 27616674 PMC5446088

[31] Wang Q, Kangas AJ, Soininen P, et al Sex hormone-binding globulin associations with circulating lipids and metabolites and the risk for type 2 diabetes: observational and causal effect estimates. Int J Epidemiol 2015;44:623–37. 10.1093/ije/dyv093 26050255

[32] Hogeveen KN, Talikka M, Hammond GL Human sex hormone-binding globulin promoter activity is influenced by a (TAAAA)n repeat element within an Alu sequence. J Biol Chem 2001;276:36383–90. 10.1074/jbc.M104681200 11473114

[33] Apter D, Bolton NJ, Hammond GL, et al Serum sex hormone-binding globulin during puberty in girls and in different types of adolescent menstrual cycles. Acta Endocrinol 1984;107:413–9. 10.1530/acta.0.1070413 6391059

[34] Norjavaara E, Ankarberg C, Albertsson-Wikland K Diurnal rhythm of 17 beta-estradiol secretion throughout pubertal development in healthy girls: evaluation by a sensitive radioimmunoassay. J Clin Endocrinol Metab 1996;81:4095–102. 10.1210/jcem.81.11.8923866 8923866

[35] Canguven O, Albayrak S Do low testosterone levels contribute to the pathogenesis of asthma? Med Hypotheses 2011;76:585–8. 10.1016/j.mehy.2011.01.006 21282014

[36] Arathimos R, Granell R, Henderson J, et al Sex discordance in asthma and wheeze prevalence in two longitudinal cohorts. PLoS One 2017;12:e0176293 10.1371/journal.pone.0176293 28441402 PMC5404857

[37] Lawlor DA, Tilling K, Davey Smith G Triangulation in aetiological epidemiology. Int J Epidemiol 2016;45:1866–86. 10.1093/ije/dyw314 28108528 PMC5841843

[38] Taieb J, Mathian B, Millot F, et al Testosterone measured by 10 immunoassays and by isotope-dilution gas chromatography-mass spectrometry in sera from 116 men, women, and children. Clin Chem 2003;49:1381–95. 10.1373/49.8.1381 12881456

[39] Burgess S, Freitag DF, Khan H, et al Using multivariable Mendelian randomization to disentangle the causal effects of lipid fractions. PLoS One 2014;9:e108891 10.1371/journal.pone.0108891 25302496 PMC4193746

