# Supplementary material for: Genetic and observational evidence supports a causal role of sex hormones on the development of asthma
Source: Thorax. 2019 Apr 1;74(7):633–42. doi: 10.1136/thoraxjnl-2018-212207 (PMC6585308; doi:10.1136/thoraxjnl-2018-212207)
Supplement: Supplementary data [file thoraxjnl-2018-212207supp001.pdf]

Supplementary Methods

ALSPAC

Study participants

ALSPAC is a large, prospective cohort study based in the South West of England[1, 2]. ALSPAC recruited 14,541 pregnant women resident in Avon, UK with expected dates of delivery 1st April 1991 to 31st December 1992. The initial number of pregnancies was 14,541 for which the mother enrolled in the ALSPAC study and had either returned at least one questionnaire or attended a "Children in Focus" clinic by 19/07/99. Of these initial pregnancies, there was a total of 14,676 fetuses, resulting in 14,062 live births and 13,988 children who were alive at 1 year of age.

When the oldest children were approximately 7 years of age, an attempt was made to bolster the initial sample with eligible cases who had failed to join the study originally. As a result, when considering variables collected from the age of seven onwards (and potentially abstracted from obstetric notes) there are data available for more than the 14,541 pregnancies mentioned above.

The number of new pregnancies not in the initial sample (known as Phase I enrolment) that are currently represented on the built files and reflecting enrolment status at the age of 18 is 706 (452 and 254 recruited during Phases II and III respectively), resulting in an additional 713 children being enrolled. The phases of enrolment are described in more detail in the cohort profile[1]. The total sample size for analyses using any data collected after the age of seven is therefore 15,247 pregnancies, resulting in 15,458 fetuses. Of this total sample of 15,458 fetuses, 14,775 were live births and 14,701 were alive at 1 year of age. Please note that the study website contains details of all the data that is available through a fully searchable data dictionary: http://www.bris.ac.uk/alspac/researchers/data-access/data-dictionary/

Written informed consent has been obtained for all ALSPAC participants. Ethical approval for the study was obtained from the ALSPAC Ethics and Law Committee and the Local Research Ethics Committees.

Sex hormones

SHBG and total testosterone were measured in peripheral blood in a subset of 513 males (512 singletons) in ALSPAC[3] . Enzyme-linked immunosorbent assays were used to measure plasma concentrations of SHBG and total testosterone in blood samples, (lithium heparin plasma), using commercially available kits. Male total testosterone measures were standardized by time of venepuncture (since testosterone displays a circadian rhythm) using multilevel modelling (in order to predict testosterone at a standard time of day), as described previously [3]. Separate models were fitted for each time-point as the effect of time of venepuncture varied by age. Age was included as a continuous variable in these models. The time-corrected values of total testosterone were those used in all analyses.

Measures of bioavailable testosterone were derived from measures of total testosterone (not corrected for time of venepuncture or exact age) and SHBG as previously described by Khairullah *et al*. [3]. Briefly, the equation "Total testosterone= Free testosterone + SHBG-bound testosterone + albumin-bound testosterone" was used, reliant on measures of SHBG assayed from the same samples and estimated concentrations of albumin-bound testosterone from a previously described reference sample[4]. The algorithm has been previously reviewed and shown strong correlations with assayed measures of bioavailable testosterone[5].

Genotypes

Genetic data for the ALSPAC children were generated by Sample Logistics and Genotyping Facilities at the Wellcome Trust Sanger Institute and LabCorp (Laboratory Corporation of America) using support from 23andMe with the Illumina Human Hap 550-quad and the Illumina GenomeStudio calling algorithm. SNPs with more than 5% of missingness, a Hardy-Weinberg-Equilibrium P-value lower than 10^{-6} or a minor allele frequency of less than 1% were removed during Quality Control (QC). Samples with indeterminate X chromosome heterozygosity or extreme autosomal heterozygosity were also excluded. SNP imputation was carried out against the 1000 Genomes Project database (www.1000genomes.org). The first 20 Principal Components (PCs) were calculated using 1.1 million HapMap3 tag SNPs.

Respiratory phenotypes

Asthma data was extracted from questionnaires completed by the mothers of the study participants at 10.7, 13.1 and 13.8 years of age, and by the study participants themselves at 16.5 and 22.9 years of age. Both the mothers and the study participants were asked the same question about asthma in the last 12 months, except at 22.9 years when study participants were asked only about ever asthma, wheeze in the last 12 months and if they had taken asthma medications in the last 12 months[(6)]. For this time point, a measure of current asthma was derived from the responses with current asthma defined as a 'yes' answer to ever asthma and a 'yes' answer to either wheeze symptoms in the last 12 months or asthma medications in the last 12 months. The reference group consisted of those who had responded 'no' to all three questions. Additionally, data on wheeze in the last 12 months were extracted from responses to questions about wheeze in the last 12 months from questionnaires completed by the mothers of the study children at an average age of 10.6, 13 and 13.8 years, and from the study children themselves at 16.5 and 18.6 years.

UK Biobank

UK Biobank received ethical approval from the Research Ethics Committee (REC reference 11/NW/0382).

Genotypes

For UK Biobank, genotypes were assayed using two different arrays, the Affymetrix UK BiLEVE Axiom or Affymetrix UK Biobank Axiom array. Imputation of genetic variants from the Haplotype Reference Consortium 3 (HRC) was also carried out. Individuals who were reported as outliers based on either genotype missingness rate or heterozygosity were excluded. Individuals whose sex inferred from the genotypes did not match their self-reported sex and individuals who demonstrated sex chromosome aneuploidy were also excluded. Finally, individuals whose ancestry was not European or who demonstrated relatedness to other study participants in UK Biobank based on kinship coefficients were removed.

GWAS

GWAS of SHBG

A previous large-scale GWAS of circulating SHBG has been conducted by Coviello *et al*. in 2012[(7)]. The study was conducted in 21,791 individuals (9,390 women and 12,401 men), from 10 cohorts and validated in 7,046 individuals (4,509 women and 2,537 men) from a further 6 collections of data. Mean age for the discovery cohorts was 19-74 years, whereas for the replication cohorts it was 32-75 years. The study reported 12 genetic variants (SNPs) associated with SHBG. These variants were mapped to *SHBG* (rs12150660), *PRMT6* (rs17496332), *GCKR* (rs780093), *ZBTB10* (rs440837), *JMJD1C* (rs7910927), *SLCO1B1* (rs4149056), *NR2F2* (rs8023580), *ZNF652* (rs2411984), *TDGF3* (rs1573036), as well as 2 conditional SNPs, *LHCGR* (rs10454142) and *BAIAP2L1* (rs3779195), and one sex-specific

SNP, *UGT2B15* (rs293428). Conditional analysis at the *SHBG* gene locus identified 4 independent signals at the genome-wide significance threshold (rs12150660, rs6258, rs1641537 and rs1625895). The lead SNP of the *SHBG* locus (rs12150660) was estimated to account for ~7.8% and ~3.3% of the variation in circulating SHBG in men and women, respectively, assuming 50% heritability. For all analyses, summary statistics from the combined discovery and replication cohorts were used, except for the SNPs from the *SHBG* locus conditional analyses where summary statistics were only available from the discovery cohort (replication was not attempted).

Statistical analyses

Observational analyses

Path analysis

Path analysis (a type of multiple regression analysis) describes associations that are hypothesized to be causal. Path diagrams represent plausible causal effects between variables whilst taking the temporal relationships of exposures, outcomes and confounders into consideration, with sex hormones considered the exposure and asthma reports the outcome. Each subsequent measure of asthma from the initial measurement and each outcome measure (SHBG or testosterone) is assumed to cause the next, in a chain of causation. Compared to serial cross-sectional regression models, the path analysis introduces additional assumptions as to the hypothesised causal paths and the temporal sequence of measures, therefore describing dependencies between the variables of the dataset. The path analysis has two main analytical advantages over classical regression analysis. Firstly, it is possible to test whether the associations between dependent and independent variables are the same across the time-points (something which we didn't end up testing as the results indicated no evidence of association at any of the five time-points). Secondly, path analysis may have increased statistical power when compared to classical regression if there is incomplete data and a full information maximum likelihood (FIML) approach is used. However, since we used multiple imputation to fill in missing responses in all variables this advantage does not directly apply to our analyses.

All path analysis models were adjusted for maternal confounders; maternal smoking during pregnancy (never, temporary, throughout pregnancy), maternal education (university degree, A-levels, O-levels or lower), parity (nulliparous, multiple pregnancies), gestational age, maternal age at birth and participants age at the time of asthma measurement (as a time-varying covariate), at each of the five analysed time-points.

Multiple imputation

Multiple imputation was used to fill in missing values in the exposure, outcome and covariates, in both the cross-sectional regressions and the path analysis. The ICE procedure in STATA 14.2[8-10] was used to perform the imputation. Due to non-normality in some of the sex hormone measures, predictive mean matching (an approach that relaxes normality assumptions in the imputed measures) was used for imputation of SHBG and testosterone values. Additionally, in order to increase imputation efficiency, auxiliary wheeze variables were included in the imputation model: responses to questions about wheeze in the last 12 months from questionnaires completed by the mothers of the study children at 10.6, 13 and 13.8 years, and from the study children themselves at 16.5 and 18.6 years. All maternal confounders as well as participants age at each time-point were included in the imputation model. Each path analysis model was then fit to 100 imputed datasets and estimates were combined using Rubin's rules[11] to obtain an overall effect size and standard error. Results using the imputed dataset were compared to the complete case analysis.

Genetic analyses

Mendelian randomization

For the inverse-variance weighted (IVW) approach which was used as the primary analysis, fixed-effects estimations were used when using 3 SNPs or fewer and random-effects for 3 SNPs or more.

Supplementary Results

Table S 1 Cross-sectional associations of SHBG and asthma. Model 1 complete case analysis adjusted for age, maternal education, parity, maternal age and maternal smoking status. Model 2 complete case analysis adjusted additionally for previous asthma and hormone measurement where available. Model 3 imputed adjusted for the same covariates as Model 1. Model 4 imputed adjusted for same covariates as Model 2.

		Model 1		Model 2		Model 3		Model 4	
Age at sex hormone exposure (years)	Age at asthma outcome (years)	OR [95% CI]	N	OR [95% CI]	N	OR [95% CI]	N	OR [95% CI]	N
9.9	10.7	0.98 [0.74,1.31]	381	0.98 [0.74,1.31]	381	0.96 [0.74,1.24]	512	0.96 [0.74,1.24]	512
11.8	13.1	1.03 [0.75,1.41]	397	2.21 [0.83,5.92]	339	0.97 [0.75,1.26]	512	1.54 [0.71,3.37]	512
13.8	13.8	0.94 [0.68,1.30]	362	1.21 [0.42,3.51]	318	0.97 [0.73,1.27]	512	1.32 [0.64,2.70]	512
15.5	16.5	1.03 [0.74,1.43]	334	1.39 [0.69,2.77]	263	1.00 [0.76,1.31]	512	1.18 [0.67,2.06]	512
17.8	22.9	1.46 [1.02,2.08]	200	0.44 [0.08,2.53]	144	0.99 [0.76,1.28]	512	1.02 [0.57,1.83]	512

Table S 2 Cross-sectional associations of total testosterone and asthma. Model 1 complete case analysis adjusted for age, maternal education, parity, maternal age and maternal smoking status. Model 2 complete case analysis adjusted additionally for previous asthma and hormone measurement where available. Model 3 imputed adjusted for the same covariates as Model 1. Model 4 imputed adjusted for same covariates as Model 2.

		Model 1		Model 2		Model 3		Model 4	
Age at sex hormone exposure (years)	Age at asthma outcome (years)	OR [95% CI]	N	OR [95% CI]	N	OR [95% CI]	N	OR [95% CI]	N
9.9	10.7	1.11 [0.85,1.44]	381	1.11 [0.85,1.44]	381	1.11 [0.87,1.42]	512	1.12 [0.87,1.43]	512
11.8	13.1	1.07 [0.82,1.40]	396	0.73 [0.39,1.37]	338	0.99 [0.77,1.28]	512	0.85 [0.55,1.32]	512
13.8	13.8	0.75 [0.54,1.03]	360	0.91 [0.46,1.81]	315	0.79 [0.60,1.05]	512	0.80 [0.46,1.38]	512
15.5	16.5	0.76 [0.54,1.06]	334	0.85 [0.50,1.45]	262	0.74 [0.55,0.99]	512	0.89 [0.59,1.35]	512
17.8	22.9	0.85 [0.56,1.27]	192	0.78 [0.21,2.84]	99	0.83 [0.64,1.07]	512	0.71 [0.43,1.16]	512

Table S 3 Cross-sectional associations of bioavailable testosterone and asthma. Model 1 complete case analysis adjusted for age, maternal education, parity, maternal age and maternal smoking status. Model 2 complete case analysis adjusted additionally for previous asthma and hormone measurement where available. Model 3 imputed adjusted for the same covariates as Model 1. Model 4 imputed adjusted for same covariates as Model 2.

		Model 1		Model 2		Model 3		Model 4	
Age at sex hormone exposure (years)	Age at asthma outcome (years)	OR [95% CI]	N	OR [95% CI]	N	OR [95% CI]	N	OR [95% CI]	N
9.9	10.7	1.02 [0.76,1.37]	381	1.02 [0.76,1.37]	381	1.02 [0.79,1.31]	512	1.02 [0.77,1.34]	512
11.8	13.1	1.03 [0.77,1.38]	396	0.71 [0.30,1.64]	338	0.99 [0.76,1.29]	512	0.79 [0.47,1.34]	512
13.8	13.8	0.83 [0.60,1.15]	360	1.08 [0.52,2.26]	315	0.89 [0.67,1.17]	512	0.86 [0.48,1.55]	512
15.5	16.5	0.82 [0.60,1.13]	334	0.77 [0.45,1.33]	262	0.82 [0.62,1.08]	512	0.87 [0.56,1.35]	512
17.8	22.9	0.54 [0.34,0.86]	192	1.97 [0.53,7.26]	99	0.93 [0.72,1.22]	512	0.87 [0.54,1.41]	512

Table S 4 Results of the path analysis of SHBG, TT or BT on asthma (using multiple imputation) adjusted for previous measures of asthma and sex hormones (where available) at five time-points in a subsample of 512 males in ALSPAC.

Age at sex hormone exposure (years)	Age at asthma outcome (years)	OR† [95% CI] SHBG	N with data for SHBG (% missing) *	OR† [95% CI] total testosterone	N with data for total testosterone (% missing) *	OR† [95% CI] bioavailable testosterone	N with data for bioavailable testosterone (% missing)	N after imputation
9.9	10.7	0.96 [0.74,1.24]	381 (26)	1.12 [0.88,1.43]	381 (26)	1.02 [0.79,1.31]	381 (26)	512
11.8	13.1	0.95 [0.64,1.41]	339 (34)	0.90 [0.63,1.30]	338 (34)	0.94 [0.63,1.41]	338 (34)	512
13.8	13.8	1.11 [0.68,1.81]	318 (38)	0.96 [0.59,1.55]	315 (38)	1.02 [0.61,1.69]	315 (38)	512
15.5	16.5	1.08 [0.76,1.53]	263 (49)	0.87 [0.60,1.26]	262 (49)	0.86 [0.60,1.23]	262 (49)	512
17.8	22.9	0.86 [0.59,1.27]	144 (72)	0.77 [0.52,1.15]	99 (81)	0.99 [0.67,1.45]	99 (81)	512

* N refers to the number of individuals with complete data in the analysis of specified hormone (before multiple imputation). Percent (%) missing refers to the fraction of individuals missing some data (either asthma measurements, hormone measurements or covariates, where covariates include any previous measurement of asthma and hormone) at each time-point, that were subject to multiple imputation.

† OR for asthma per standard deviation (SD) increase in either SHBG, TT or BT.

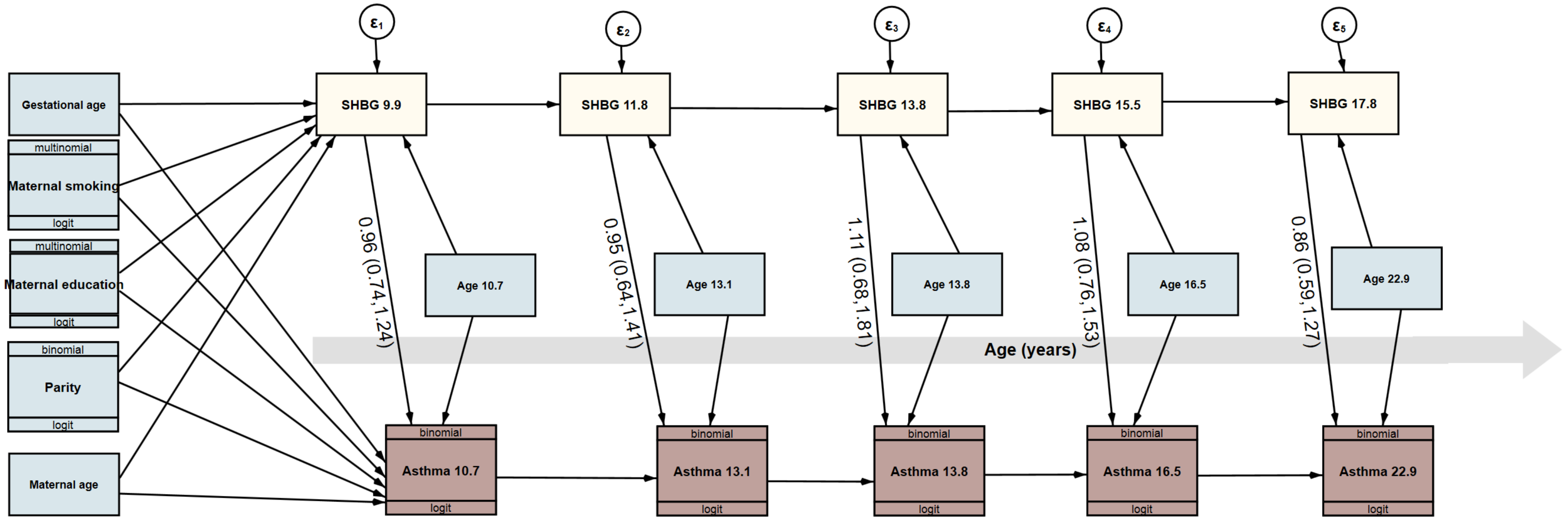

Figure S 1 Path analysis of SHBG and asthma in ALSPAC.

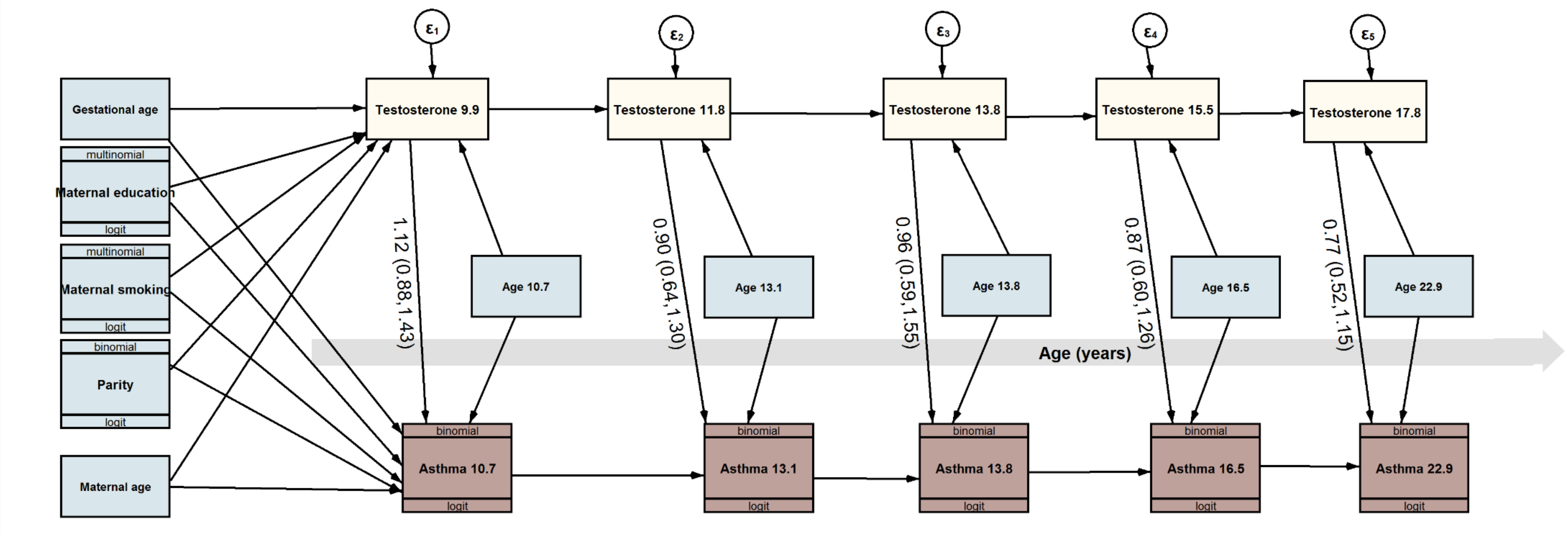

Figure S 2 Path analysis of total testosterone (TT) and asthma in ALSPAC.

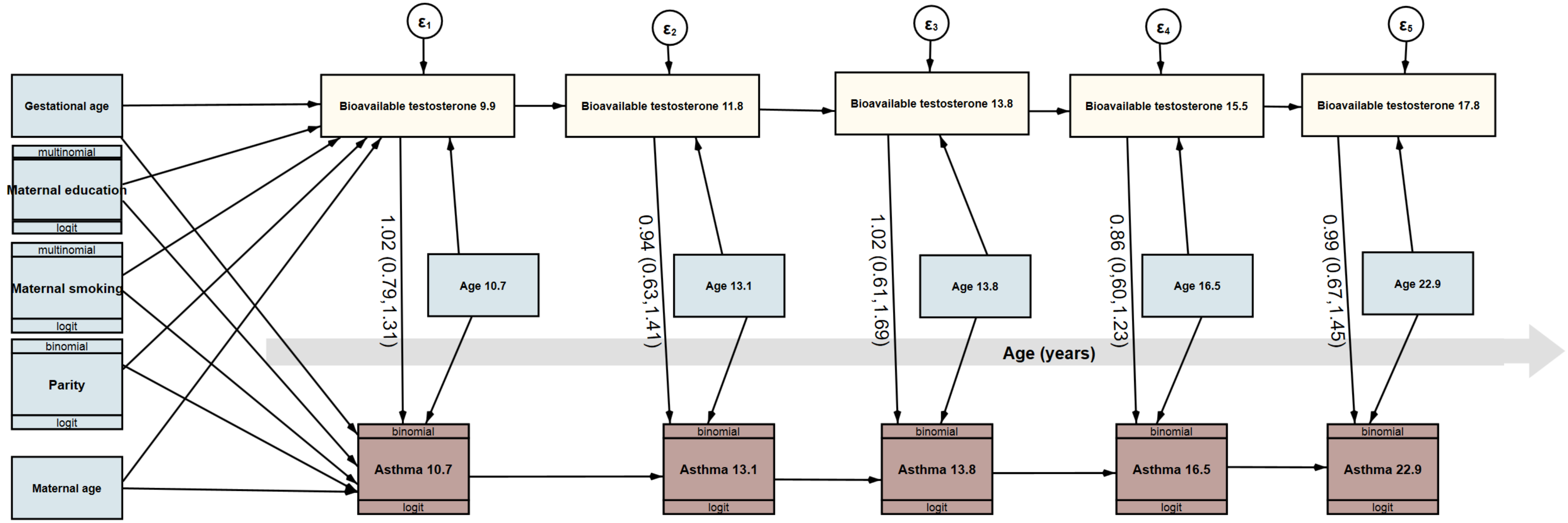

Figure S 3 Path analysis of bioavailable testosterone (BT) in ALSPAC

Table S 5 SNP-SHBG effects for IVs used in combination A as reported by Coviello et al. in the GWAS of SHBG, with calculated proportion of variance explained (PVE).

SNP	Analysis‡	Region	Gene	Chr	Position	Effect	Other	EAF	Beta†	SE†	Pvalue†	PVE*
rs17496332	Main	1p13.3	*PRMT6*	1	1.07E+08	a	g	0.67	-0.028	0.0041	1.40E-11	0.002136
rs780093	Main	2p23.3	*GCKR*	2	27596107	t	c	0.4	-0.032	0.0039	2.20E-16	0.00308
rs440837	Main	8q21.13	*ZBTB10*	8	81624529	a	g	0.78	-0.028	0.0047	3.40E-09	0.001626
rs7910927	Main	10q21.3	*JMJD1C*	10	64808916	t	g	0.51	-0.048	0.0039	6.10E-35	0.006903
rs4149056	Main	12p12.1	*SLCO1B1*	12	21222816	t	c	0.82	0.029	0.0052	1.90E-08	0.001425
rs8023580	Main	15q26.2	*NR2F2*	15	94509295	t	c	0.72	-0.03	0.0044	8.30E-12	0.002129
rs12150660	Main	17p13.1	*SHBG*	17	7462640	t	g	0.24	0.103	0.0047	1.80E-106	0.021564
rs1641537	Indep	17p13.1	*SHBG*	17	7486446	t	c	0.14	-0.064	0.006	1.20E-24	0.005194
rs1625895	Indep	17p13.1	*SHBG*	17	7518840	t	c	0.12	-0.06	0.006	1.75E-21	0.004568

**PVE – Proportion of variance explained for each SNP, calculated based on reported summary statistics and the formula by Shim et al.(12)*

†Effect estimate, standard error and p-value from the combined discovery plus follow-up analysis by Coviello et al.(7) for the whole sample, except for the independent SNP analysis where only the discovery sample was used.

‡Main GWAS analysis by Coviello et al. or independent SNPs analysis of the SHBG gene region.

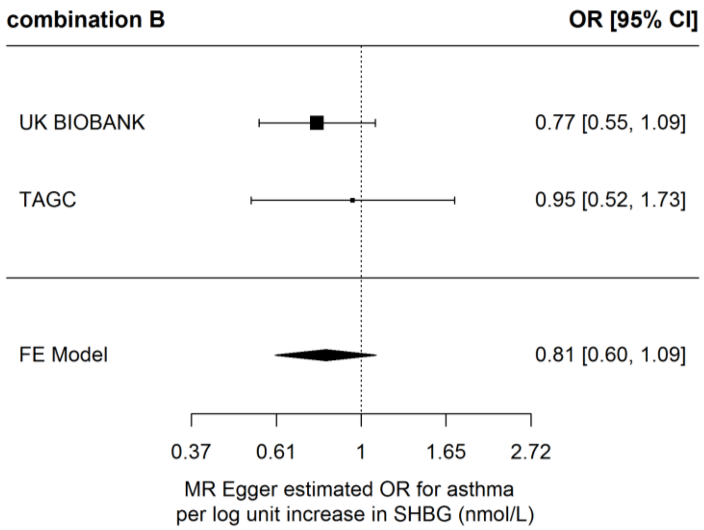

Figure S 4 Forest plot of the fixed effects meta-analysis of the MR Egger regression estimated effects of IV combination B on asthma in UK Biobank and the TAGC consortium GWAS.

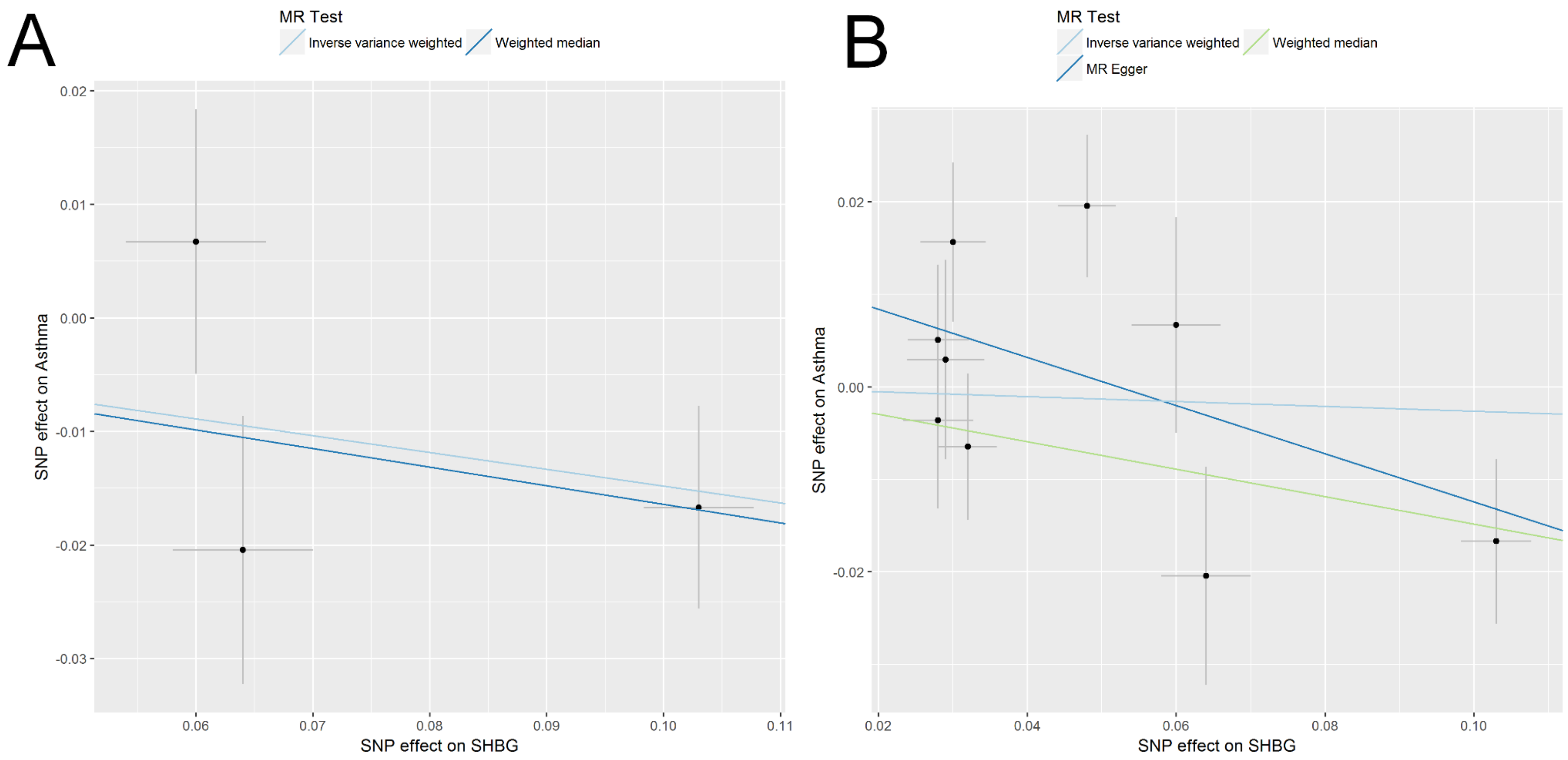

Figure S 5 Scatter plots of the SNP-asthma and SNP-SHBG effects in UK Biobank. A) SNP combination A, B) SNP combination B

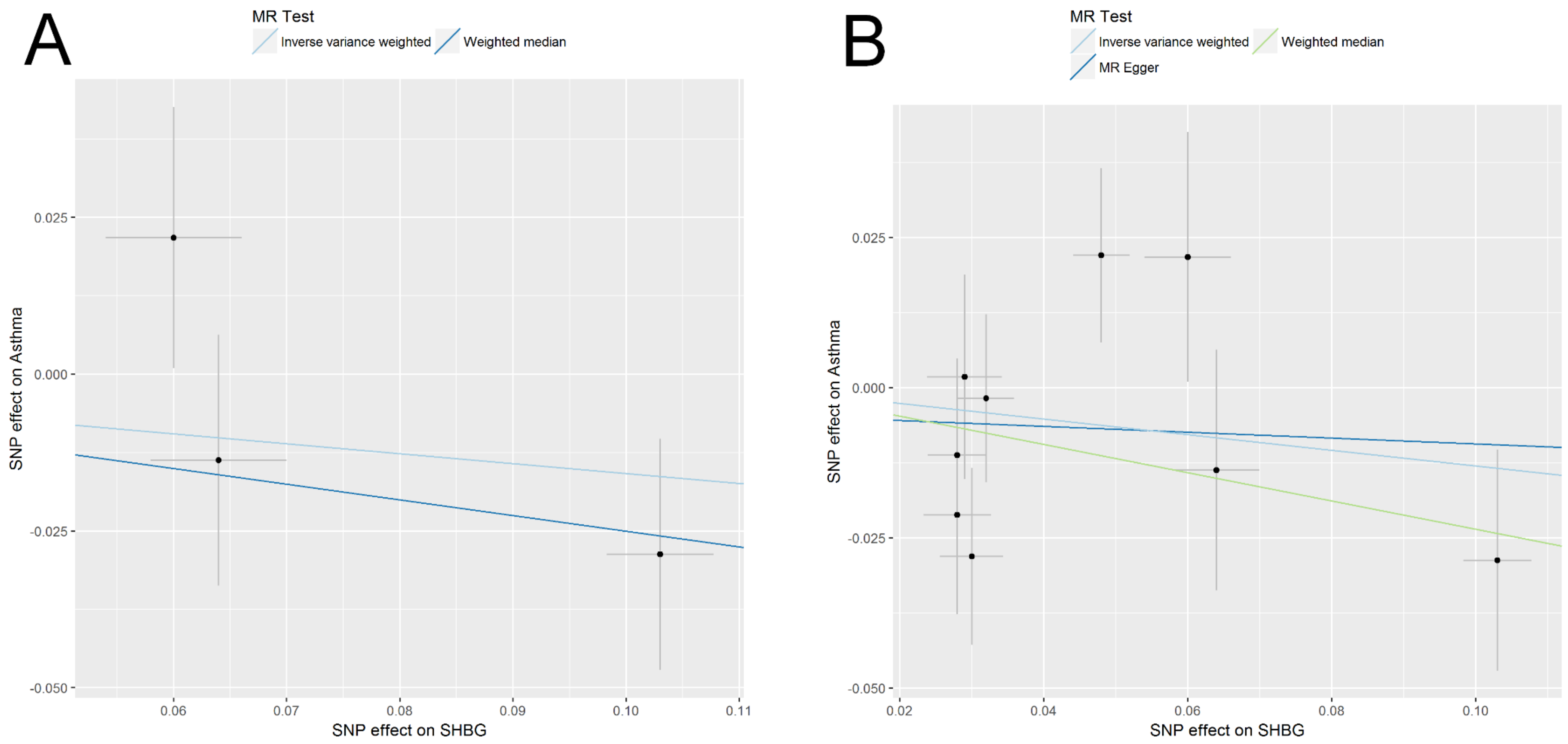

Figure S 6 Scatter plots of the SNP-asthma and SNP-SHBG effects in TAGC. A) SNP combination A, B) SNP combination B.

Table S 6 MR estimated effects from UK Biobank.

Outcome	Exposure	Method	N SNPs	Estimate†	SE	P-value	Lower 95% CI	Upper 95% CI
**Combination A**								
Asthma	SHBG	Weighted median	3	-0.16422	0.080955	0.042505	-0.32289	-0.00555
Asthma	SHBG	Inverse variance weighted	3	-0.14811	0.084089	0.078172	-0.31293	0.016701
**Combination B**								
Asthma	SHBG	MR Egger	9	-0.26026	0.174735	0.179975	-0.60274	0.082217
Asthma	SHBG	Weighted median	9	-0.14838	0.080776	0.066217	-0.3067	0.009939
Asthma	SHBG	Inverse variance weighted	9	-0.02616	0.088074	0.766469	-0.19878	0.146468

†Estimated effects are log OR for asthma per one unit increase in log SHBG

Table S 7 MR estimated effects from TAGC GWAS of asthma.

Outcome	Exposure	Method	N SNPs	Estimate†	SE	P-value	Lower 95% CI	Upper 95% CI
**Combination A**								
Asthma	SHBG	Weighted median	3	-0.25018	0.160759	0.110976	-0.55784	0.057479
Asthma	SHBG	Inverse variance weighted	3	-0.15855	0.165944	0.339344	-0.4838	0.166697
**Combination B**								
Asthma	SHBG	MR Egger	9	-0.04919	0.30558	0.87666	-0.64813	0.549746
Asthma	SHBG	Weighted median	9	-0.23574	0.154057	0.125969	-0.53769	0.066215
Asthma	SHBG	Inverse variance weighted	9	-0.13043	0.130583	0.317884	-0.38637	0.125514

† Estimated effects are log OR for asthma per one unit increase in log SHBG

Table S 8 Meta-analysed results of MR estimated effects of SHBG on asthma across UK Biobank and TAGC.

Outcome	Exposure	Method	Estimate†	SE	Lower 95% CI	Upper 95% CI
**Combination A**						
Asthma	SHBG	Weighted median	-0.182	0.072	-0.323	-0.0413
Asthma	SHBG	Inverse variance weighted	-0.150	0.075	-0.297	-0.003
**Combination B**						
Asthma	SHBG	MR Egger	-0.2083	0.152	-0.506	0.089
Asthma	SHBG	Weighted median	-0.167	0.072	-0.307	-0.027
Asthma	SHBG	Inverse variance weighted	-0.059	0.073	-0.202	0.084

† Estimated effects are log OR for asthma per one unit increase in log SHBG

Sex-stratified MR results

IV combination B

In females when using IV combination B, ORs for IVW, weighted-median and MR-Egger were 0.94 [95% CI 0.76,1.17], 0.85 [95% CI 0.69,1.05] and 0.67 [95% CI 0.44,1.02] respectively. For males, IV combination B ORs were 1.01 [95% CI 0.83,1.25] for IVW, 0.93 [95% CI 0.75,1.16] for the weighted-median estimated effect and 0.91 [95% CI 0.58,1.46] for MR Egger estimated effect. Evidence from the IV heterogeneity test (Cochranes Q = 16.3, Q p-value= 0.038) and from the MR-Egger intercept test (intercept odds=1.02, P=0.117) suggested some heterogeneity and horizontal pleiotropy in IV combination B for females. A sensitivity analysis using sex-specific SNP-SHBG effects from the sex-stratified GWAS(7) did not indicate any substantial differences in observed effects when compared to the whole-sample GWAS effects (data not shown).

Table S 9 MR estimated effects of SHBG on asthma in the female subsample of UK Biobank.

Outcome	Exposure	Method	N SNPs	Estimate†	SE	P-value	Lower 95% CI	Upper 95% CI
**Combination A**								
Asthma	SHBG	Weighted median	3	-0.22963	0.107212	0.032206	-0.43977	-0.0195
Asthma	SHBG	Inverse variance weighted	3	-0.22349	0.120146	0.062866	-0.45897	0.011998
**Combination B**								
Asthma	SHBG	MR Egger	9	-0.39505	0.212165	0.104907	-0.81089	0.020798
Asthma	SHBG	Weighted median	9	-0.16546	0.107335	0.123198	-0.37583	0.044921
Asthma	SHBG	Inverse variance weighted	9	-0.05932	0.112027	0.596445	-0.27889	0.160253

† Estimated effects are log OR for asthma per one unit increase in log SHBG

Table S 10 MR estimated effects of SHBG on asthma in the male subsample of UK Biobank.

Outcome	Exposure	Method	N SNPs	Estimate†	SE	P-value	Lower 95% CI	Upper 95% CI
**Combination A**								
Asthma	SHBG	Weighted median	3	-0.07483	0.119803	0.532253	-0.30964	0.159989
Asthma	SHBG	Inverse variance weighted	3	-0.05084	0.11084	0.64645	-0.26809	0.166404
**Combination B**								
Asthma	SHBG	MR Egger	9	-0.08549	0.236467	0.728378	-0.54896	0.377988
Asthma	SHBG	Weighted median	9	-0.0697	0.109827	0.52566	-0.28496	0.14556
Asthma	SHBG	Inverse variance weighted	9	0.013952	0.105092	0.894382	-0.19203	0.219932

† Estimated effects are log OR for asthma per one unit increase in log SHBG

References

1. Boyd A, Golding J, Macleod J, Lawlor DA, Fraser A, Henderson J. Cohort profile: the 'children of the 90s'--the index offspring of the Avon Longitudinal Study of Parents and Children. Int J Epidemiol. 2013;42.
2. Fraser A, Macdonald-Wallis C, Tilling K, Boyd A, Golding J, Davey Smith G. Cohort profile: the Avon Longitudinal Study of Parents and Children: ALSPAC mothers cohort. Int J Epidemiol. 2013;42.
3. Khairullah A, Cousino Klein L, Ingle SM, May MT, Whetzel CA, Susman EJ, et al. Testosterone Trajectories and Reference Ranges in a Large Longitudinal Sample of Male Adolescents. PLoS ONE. 2014;9(9):e108838.
4. Sodergard R, Backstrom T, Shanbhag V, Carstensen H. Calculation of free and bound fractions of testosterone and estradiol-17 beta to human plasma proteins at body temperature. Journal of steroid biochemistry. 1982;16(6):801-10.
5. Emadi-Konjin P, Bain J, Bromberg IL. Evaluation of an algorithm for calculation of serum "Bioavailable" testosterone (BAT). Clinical Biochemistry. 2003;36(8):591-6.
6. Harris PA, Taylor R, Thielke R, Payne J, Gonzalez N, Conde JG. Research electronic data capture (REDCap)—A metadata-driven methodology and workflow process for providing translational research informatics support. Journal of Biomedical Informatics. 2009;42(2):377-81.
7. Coviello AD, Haring R, Wellons M, Vaidya D, Lehtimäki T, Keildson S, et al. A genome-wide association meta-analysis of circulating sex hormone-binding globulin reveals multiple Loci implicated in sex steroid hormone regulation. PLoS genetics. 2012;8(7):e1002805.
8. Royston P. ICE: Stata module for multiple imputation of missing values. Statistical Software Components. 2014.
9. Royston P, White IR. Multiple imputation by chained equations (MICE): implementation in Stata. Journal of Statistical Software. 2011;45(4):1-20.
10. StataCorp. Stata Statistical Software: Release 14. College Station, TX: StataCorp LP. 2015.
11. Rubin DB. Multiple Imputation for Nonresponse in Surveys. New York, NY: Wiley; 1987.
12. Shim H, Chasman DI, Smith JD, Mora S, Ridker PM, Nickerson DA, et al. A Multivariate Genome-Wide Association Analysis of 10 LDL Subfractions, and Their Response to Statin Treatment, in 1868 Caucasians. PLOS ONE. 2015;10(4):e0120758.
