# Supplementary material for: Genetic and observational evidence supports a causal role of sex hormones on the development of asthma
Source: Thorax. 2019 Apr 1;74(7):633–42. doi: 10.1136/thoraxjnl-2018-212207 (PMC6585308; doi:10.1136/thoraxjnl-2018-212207)
Supplement: Supplementary data [file thoraxjnl-2018-212207supp002.pdf]

SNP (SHBG raising allele)	Outcome	Age	Estimate [95%CI]	std.error	p.value	N
rs17496332_G	SHBG	9.9	0.0525 [-0.108 , 0.213]	0.082042	0.522336	374
rs17496332_G	SHBG	11.8	0.00735 [-0.147 , 0.162]	0.078966	0.925905	410
rs17496332_G	SHBG	13.8	-0.00952 [-0.184 , 0.165]	0.08925	0.915138	351
rs17496332_G	SHBG	15.5	0.00184 [-0.157 , 0.161]	0.081251	0.981988	396
rs17496332_G	SHBG	17.8	-0.00511 [-0.167 , 0.157]	0.082786	0.950798	368
rs17496332_G	Bioavailable Testosterone	9.9	-0.00707 [-0.169 , 0.154]	0.082382	0.931662	373
rs17496332_G	Bioavailable Testosterone	11.8	0.000132 [-0.156 , 0.157]	0.079839	0.998684	410
rs17496332_G	Bioavailable Testosterone	13.8	0.118 [-0.0565 , 0.292]	0.088904	0.186159	349
rs17496332_G	Bioavailable Testosterone	15.5	0.0294 [-0.13 , 0.189]	0.081308	0.717918	396
rs17496332_G	Bioavailable Testosterone	17.8	0.00654 [-0.158 , 0.171]	0.083863	0.93793	357
rs17496332_G	Total Testosterone	9.9	0.0116 [-0.148 , 0.172]	0.081605	0.887255	373
rs17496332_G	Total Testosterone	11.8	0.0102 [-0.146 , 0.166]	0.079567	0.898232	410
rs17496332_G	Total Testosterone	13.8	0.0734 [-0.102 , 0.249]	0.089345	0.411962	349
rs17496332_G	Total Testosterone	15.5	0.0324 [-0.127 , 0.192]	0.081381	0.690486	396
rs17496332_G	Total Testosterone	17.8	-0.00324 [-0.165 , 0.159]	0.082728	0.968799	357
rs440837_G	SHBG	9.9	0.14 [-0.0337 , 0.314]	0.088693	0.115031	374
rs440837_G	SHBG	11.8	0.0534 [-0.114 , 0.221]	0.085326	0.531917	410
rs440837_G	SHBG	13.8	0.22 [0.0362 , 0.405]	0.094004	0.019621	351
rs440837_G	SHBG	15.5	0.0551 [-0.111 , 0.221]	0.084711	0.516111	396
rs440837_G	SHBG	17.8	0.0396 [-0.135 , 0.214]	0.08898	0.656325	368
rs440837_G	Bioavailable Testosterone	9.9	-0.133 [-0.308 , 0.0411]	0.089016	0.134935	373
rs440837_G	Bioavailable Testosterone	11.8	-0.0633 [-0.232 , 0.106]	0.086252	0.463581	410
rs440837_G	Bioavailable Testosterone	13.8	-0.177 [-0.361 , 0.00814]	0.094265	0.061867	349
rs440837_G	Bioavailable Testosterone	15.5	0.0809 [-0.0852 , 0.247]	0.08473	0.340427	396
rs440837_G	Bioavailable Testosterone	17.8	-2.81e-05 [-0.177 , 0.177]	0.090217	0.999752	357
rs440837_G	Total Testosterone	9.9	0.0153 [-0.158 , 0.189]	0.088456	0.863088	373
rs440837_G	Total Testosterone	11.8	-0.0777 [-0.246 , 0.0907]	0.085929	0.366212	410
rs440837_G	Total Testosterone	13.8	-0.128 [-0.314 , 0.058]	0.09482	0.178584	349
rs440837_G	Total Testosterone	15.5	0.125 [-0.0412 , 0.291]	0.084667	0.141443	396
rs440837_G	Total Testosterone	17.8	-0.00722 [-0.182 , 0.167]	0.088994	0.935414	357
rs7910927_G	SHBG	9.9	0.0258 [-0.118 , 0.169]	0.073136	0.724254	374
rs7910927_G	SHBG	11.8	-0.0144 [-0.151 , 0.123]	0.069913	0.83689	410

rs7910927_G	SHBG	13.8	0.0928 [-0.0578 , 0.243]	0.076811	0.227875	351
rs7910927_G	SHBG	15.5	-0.0697 [-0.211 , 0.0713]	0.071933	0.332972	396
rs7910927_G	SHBG	17.8	0.101 [-0.0452 , 0.247]	0.074413	0.177033	368
rs7910927_G	Bioavailable Testosterone	9.9	-0.0506 [-0.195 , 0.0936]	0.073565	0.492434	373
rs7910927_G	Bioavailable Testosterone	11.8	-0.0444 [-0.183 , 0.0941]	0.070653	0.530152	410
rs7910927_G	Bioavailable Testosterone	13.8	-0.0653 [-0.216 , 0.0854]	0.076874	0.396204	349
rs7910927_G	Bioavailable Testosterone	15.5	0.0571 [-0.084 , 0.198]	0.072026	0.428096	396
rs7910927_G	Bioavailable Testosterone	17.8	0.019 [-0.128 , 0.166]	0.075079	0.800173	357
rs7910927_G	Total Testosterone	9.9	0.00581 [-0.137 , 0.149]	0.072921	0.936553	373
rs7910927_G	Total Testosterone	11.8	-0.0299 [-0.168 , 0.108]	0.070434	0.671155	410
rs7910927_G	Total Testosterone	13.8	-0.0406 [-0.192 , 0.111]	0.07718	0.598965	349
rs7910927_G	Total Testosterone	15.5	0.0495 [-0.0918 , 0.191]	0.072109	0.492556	396
rs7910927_G	Total Testosterone	17.8	0.109 [-0.0358 , 0.254]	0.07383	0.141076	357
rs4149056_T	SHBG	9.9	0.00763 [-0.196 , 0.211]	0.103713	0.941368	374
rs4149056_T	SHBG	11.8	-0.0139 [-0.209 , 0.181]	0.099284	0.888466	410
rs4149056_T	SHBG	13.8	0.09 [-0.129 , 0.309]	0.111607	0.420474	351
rs4149056_T	SHBG	15.5	0.102 [-0.0932 , 0.297]	0.099628	0.306055	396
rs4149056_T	SHBG	17.8	0.119 [-0.0852 , 0.322]	0.10394	0.254845	368
rs4149056_T	Bioavailable Testosterone	9.9	0.0801 [-0.124 , 0.284]	0.104079	0.441915	373
rs4149056_T	Bioavailable Testosterone	11.8	0.0261 [-0.171 , 0.223]	0.100374	0.794621	410
rs4149056_T	Bioavailable Testosterone	13.8	-0.053 [-0.272 , 0.166]	0.111504	0.634969	349
rs4149056_T	Bioavailable Testosterone	15.5	0.0829 [-0.113 , 0.278]	0.099764	0.406739	396
rs4149056_T	Bioavailable Testosterone	17.8	0.0292 [-0.176 , 0.234]	0.104518	0.779818	357
rs4149056_T	Total Testosterone	9.9	0.169 [-0.0322 , 0.371]	0.10279	0.100567	373
rs4149056_T	Total Testosterone	11.8	0.0787 [-0.117 , 0.275]	0.099964	0.431416	410
rs4149056_T	Total Testosterone	13.8	0.0015 [-0.218 , 0.221]	0.111911	0.989302	349
rs4149056_T	Total Testosterone	15.5	0.197 [0.00249 , 0.392]	0.099427	0.047873	396
rs4149056_T	Total Testosterone	17.8	0.136 [-0.0661 , 0.337]	0.102848	0.188544	357
rs8023580_C	SHBG	9.9	0.228 [0.0595 , 0.396]	0.085766	0.00833	374
rs8023580_C	SHBG	11.8	0.0842 [-0.0783 , 0.247]	0.08292	0.310555	410
rs8023580_C	SHBG	13.8	0.045 [-0.135 , 0.225]	0.091767	0.624321	351
rs8023580_C	SHBG	15.5	0.00863 [-0.155 , 0.172]	0.083294	0.917498	396
rs8023580_C	SHBG	17.8	0.0845 [-0.0869 , 0.256]	0.087474	0.334709	368

rs8023580_C	Bioavailable Testosterone	9.9	-0.243 [-0.411 , -0.0743]	0.085951	0.005013	373
rs8023580_C	Bioavailable Testosterone	11.8	0.0231 [-0.141 , 0.188]	0.083939	0.782885	410
rs8023580_C	Bioavailable Testosterone	13.8	-0.012 [-0.192 , 0.168]	0.091877	0.895824	349
rs8023580_C	Bioavailable Testosterone	15.5	0.0112 [-0.152 , 0.175]	0.083366	0.893485	396
rs8023580_C	Bioavailable Testosterone	17.8	-0.141 [-0.315 , 0.0317]	0.088312	0.110194	357
rs8023580_C	Total Testosterone	9.9	0.0836 [-0.0849 , 0.252]	0.085988	0.33153	373
rs8023580_C	Total Testosterone	11.8	0.0892 [-0.0746 , 0.253]	0.08354	0.286367	410
rs8023580_C	Total Testosterone	13.8	-0.00178 [-0.182 , 0.179]	0.092183	0.984572	349
rs8023580_C	Total Testosterone	15.5	0.072 [-0.0914 , 0.235]	0.083363	0.388569	396
rs8023580_C	Total Testosterone	17.8	-0.0755 [-0.247 , 0.0957]	0.087352	0.387828	357
rs780093_C	SHBG	9.9	0.094 [-0.0558 , 0.244]	0.076458	0.219652	374
rs780093_C	SHBG	11.8	0.124 [-0.0212 , 0.269]	0.074094	0.095053	410
rs780093_C	SHBG	13.8	0.0367 [-0.126 , 0.199]	0.083047	0.659094	351
rs780093_C	SHBG	15.5	0.033 [-0.117 , 0.183]	0.076602	0.666598	396
rs780093_C	SHBG	17.8	0.0529 [-0.0979 , 0.204]	0.076944	0.492098	368
rs780093_C	Bioavailable Testosterone	9.9	-0.0974 [-0.248 , 0.0534]	0.076942	0.206509	373
rs780093_C	Bioavailable Testosterone	11.8	-0.117 [-0.264 , 0.0297]	0.074946	0.118684	410
rs780093_C	Bioavailable Testosterone	13.8	0.0161 [-0.148 , 0.18]	0.083521	0.847347	349
rs780093_C	Bioavailable Testosterone	15.5	0.0139 [-0.136 , 0.164]	0.076685	0.855971	396
rs780093_C	Bioavailable Testosterone	17.8	-0.0811 [-0.234 , 0.0723]	0.07825	0.300968	357
rs780093_C	Total Testosterone	9.9	0.115 [-0.0339 , 0.265]	0.076143	0.130797	373
rs780093_C	Total Testosterone	11.8	-0.105 [-0.252 , 0.0413]	0.074737	0.16025	410
rs780093_C	Total Testosterone	13.8	0.0591 [-0.105 , 0.223]	0.083739	0.481111	349
rs780093_C	Total Testosterone	15.5	0.0856 [-0.0646 , 0.236]	0.076632	0.264823	396
rs780093_C	Total Testosterone	17.8	0.0403 [-0.111 , 0.192]	0.077283	0.602574	357
rs12150660_T	SHBG	9.9	0.411 [0.235 , 0.586]	0.089482	6.16E-06	374
rs12150660_T	SHBG	11.8	0.389 [0.226 , 0.552]	0.083364	4.23E-06	410
rs12150660_T	SHBG	13.8	0.236 [0.057 , 0.416]	0.091447	0.010203	351
rs12150660_T	SHBG	15.5	0.377 [0.208 , 0.545]	0.085864	1.50E-05	396
rs12150660_T	SHBG	17.8	0.375 [0.203 , 0.546]	0.08759	2.44E-05	368
rs12150660_T	Bioavailable Testosterone	9.9	-0.365 [-0.542 , -0.188]	0.090387	6.68E-05	373
rs12150660_T	Bioavailable Testosterone	11.8	-0.149 [-0.318 , 0.0202]	0.086287	0.08516	410
rs12150660_T	Bioavailable Testosterone	13.8	0.0992 [-0.0817 , 0.28]	0.092296	0.283298	349

rs12150660_T	Bioavailable Testosterone	15.5	-0.0985 [-0.271 , 0.0739]	0.087975	0.263634	396
rs12150660_T	Bioavailable Testosterone	17.8	-0.228 [-0.404 , -0.0518]	0.089872	0.011644	357
rs12150660_T	Total Testosterone	9.9	-0.0273 [-0.207 , 0.152]	0.091579	0.76614	373
rs12150660_T	Total Testosterone	11.8	-0.0685 [-0.238 , 0.101]	0.086254	0.427483	410
rs12150660_T	Total Testosterone	13.8	0.272 [0.0924 , 0.451]	0.091538	0.003207	349
rs12150660_T	Total Testosterone	15.5	0.299 [0.128 , 0.469]	0.086844	0.000653	396
rs12150660_T	Total Testosterone	17.8	0.304 [0.131 , 0.476]	0.087951	0.000624	357
rs1641537_C	SHBG	9.9	0.356 [0.151 , 0.56]	0.104361	0.000725	374
rs1641537_C	SHBG	11.8	0.286 [0.088 , 0.484]	0.101149	0.004899	410
rs1641537_C	SHBG	13.8	0.158 [-0.0652 , 0.38]	0.113651	0.166699	351
rs1641537_C	SHBG	15.5	0.122 [-0.0814 , 0.326]	0.103994	0.239796	396
rs1641537_C	SHBG	17.8	0.103 [-0.115 , 0.32]	0.110857	0.354554	368
rs1641537_C	Bioavailable Testosterone	9.9	-0.453 [-0.657 , -0.25]	0.103785	1.65E-05	373
rs1641537_C	Bioavailable Testosterone	11.8	-0.151 [-0.353 , 0.0511]	0.103032	0.144072	410
rs1641537_C	Bioavailable Testosterone	13.8	-0.0813 [-0.305 , 0.142]	0.114085	0.476651	349
rs1641537_C	Bioavailable Testosterone	15.5	0.0214 [-0.183 , 0.226]	0.104272	0.837707	396
rs1641537_C	Bioavailable Testosterone	17.8	-0.171 [-0.39 , 0.0484]	0.111895	0.127517	357
rs1641537_C	Total Testosterone	9.9	-0.188 [-0.394 , 0.0181]	0.105088	0.074747	373
rs1641537_C	Total Testosterone	11.8	0.00117 [-0.201 , 0.203]	0.102966	0.990961	410
rs1641537_C	Total Testosterone	13.8	0.0884 [-0.136 , 0.313]	0.114447	0.44059	349
rs1641537_C	Total Testosterone	15.5	0.22 [0.0167 , 0.423]	0.103753	0.034591	396
rs1641537_C	Total Testosterone	17.8	0.0683 [-0.149 , 0.285]	0.110701	0.537708	357
rs1625895_C	SHBG	9.9	0.178 [-0.0847 , 0.441]	0.134134	0.184822	374
rs1625895_C	SHBG	11.8	-0.0542 [-0.316 , 0.208]	0.133583	0.685201	410
rs1625895_C	SHBG	13.8	0.055 [-0.224 , 0.334]	0.142224	0.699055	351
rs1625895_C	SHBG	15.5	0.282 [0.0242 , 0.54]	0.131693	0.032717	396
rs1625895_C	SHBG	17.8	0.0825 [-0.176 , 0.341]	0.131744	0.531405	368
rs1625895_C	Bioavailable Testosterone	9.9	-0.0901 [-0.354 , 0.174]	0.134744	0.504291	373
rs1625895_C	Bioavailable Testosterone	11.8	0.241 [-0.0224 , 0.505]	0.13453	0.073639	410
rs1625895_C	Bioavailable Testosterone	13.8	0.0844 [-0.194 , 0.363]	0.141914	0.552471	349
rs1625895_C	Bioavailable Testosterone	15.5	-0.26 [-0.518 , -0.000922]	0.131935	0.049923	396
rs1625895_C	Bioavailable Testosterone	17.8	-0.00648 [-0.266 , 0.253]	0.132378	0.960998	357
rs1625895_C	Total Testosterone	9.9	0.0626 [-0.199 , 0.324]	0.133518	0.639702	373

rs1625895_C	Total Testosterone	11.8	0.217 [-0.0462 , 0.48]	0.134179	0.106916	410
rs1625895_C	Total Testosterone	13.8	0.071 [-0.208 , 0.35]	0.142406	0.618563	349
rs1625895_C	Total Testosterone	15.5	-0.0255 [-0.286 , 0.235]	0.132733	0.847765	396
rs1625895_C	Total Testosterone	17.8	0.123 [-0.133 , 0.378]	0.130414	0.347641	357
